# Maternal Overweight/Obesity and Polyunsaturated Fatty Acid Status During Pregnancy Are Associated with Offspring Body Composition at 8.5 Years: Findings from the PREOBE Study

**DOI:** 10.3390/nu18203453

**Published:** 2026-10-21

**Authors:** Mireia Escudero-Marín, Hatim Azaryah, Andrea De La Garza, Carmen López-Sabater, Rocío Bonillo-León, Luz María García-Valdés, Elvira Verduci, Cristina Campoy

**Affiliations:** 1 Department of Pediatrics, School of Medicine, University of Granada, Avda. Jesús Candel Fábregas, 18016 Granada, Spain; m.escuderomarin@gmail.com (M.E.-M.);; 2 EURISTIKOS Excellence Centre for Pediatric Research, University of Granada, Avda. del Conocimiento, 19, 18016 Granada, Spain; 3 Instituto de Investigación Biosanitaria de Granada (Ibs-GRANADA), Avda. de Madrid, 15, Beiro, 18012 Granada, Spain; 4 Institute of Neurosciences, Biomedical Research Centre, University of Granada, Avda. del Conocimiento, 19, 18012 Granada, Spain; 5 Department of Nutrition, Food Sciences and Gastronomy, XIA School of Pharmacy and Food Sciences, University of Barcelona, 08007 Barcelona, Spain; 6 Institut de Recerca en Nutrició i Seguretat Alimentària UB (INSA-UB), 08921 Barcelona, Spain; 7 CIBER Physiopathology of Obesity and Nutrition CIBERobn, Institute of Health Carlos III, 28029 Madrid, Spain; 8 Department of Preventive Medicine and Public Health, Faculty of Medicine, University of Granada, 18012 Granada, Spain; 9 CIBER on Epidemiology and Public Health CIBEResp, Institute of Health Carlos III, 28029 Madrid, Spain; 10 Pediatric Unit, Foundation IRCCS Ca’ Granda Ospedale Maggiore Policlinico, 20122 Milan, Italy; 11 Department of Health Sciences, University of Milan, 20122 Milan, Italy

**Keywords:** pregnancy in obesity, fatty acids, polyunsaturated fatty acids, childhood body composition, arachidonic acid (ARA), docosahexaenoic acid (DHA), gamma-linolenic acid (GLA), dihomo-gamma-linolenic acid (DGLA), *n*-6 PUFAs, *n*-3 PUFAs, developmental programming

## Abstract

Background: Maternal obesity is associated with altered polyunsaturated fatty acid (PUFA) status during pregnancy and may programme offspring adiposity, yet the contribution of specific *n*-3 and *n*-6 PUFAs across gestation and in cord blood to childhood body composition remains incompletely understood. We investigated these associations between maternal PUFA status during pregnancy and offspring obesity-related outcomes at 8.5 years. Methods: Here, we analysed data of 172 mother–child pairs from the PREOBE cohort, grouped by maternal pregestational body mass index (BMI) as normal weight (NW, n = 84), overweight (OW, n = 47), or obese (OB, n = 41). Plasma phospholipid PUFAs were quantified at 24, 34, and 40 weeks of gestation and in cord blood. At 8.5 years, children underwent anthropometry and body-composition assessment by skinfolds and bioelectrical impedance, remaining 104 mother–child pairs with valid data. Associations analyses included ANOVA/ANCOVA adjusted by confounders variables, unadjusted bivariate Spearman rank correlations with Benjamini–Hochberg False Discovery Rate (FDR) correction, and pooled robust linear regression. Results: Compared with NW mothers, OW and OB mothers showed lower α-linolenic acid (ALA) and docosahexaenoic acid (DHA) and higher *n*-6 PUFAs and *n*-6/*n*-3 ratios during pregnancy. Cord γ-linolenic acid (GLA) was inversely associated with several adiposity measures in the NW and OW groups after FDR correction. No correlation in the OB group met the FDR threshold; the inverse cord GLA–fat-free mass index (FFMI) association was nominal only (ρ = −0.52, *p* = 0.016, q = 0.688). In pooled regression models, cord GLA was inversely associated with BMI-for-age z-score, waist circumference z-score, waist-to-height ratio, body fat mass percentage, and fat mass index (FMI), whereas maternal GLA at 40 weeks was positively associated with body fat mass percentage and FMI. These pooled estimates do not establish group-specific effects. Conclusions: Maternal and cord-blood PUFA profiles were associated with childhood body composition, with patterns that varied descriptively by sampling time and maternal BMI group. These observational findings do not establish causality, protection, or differences between groups in the strength of association. Mechanistic and interventional research is required before individualised long-chain PUFA monitoring or supplementation can be recommended for obesity prevention.

## 1. Introduction

The Developmental Origins of Health and Disease (DOHaD) hypothesis postulates that nutritional exposures during critical pre- and postnatal windows can programme metabolic trajectories, increasing the risk of chronic disease later in life [1,2]. Childhood overweight and obesity remain substantial public health concerns: the World Health Organization (WHO) European Childhood Obesity Surveillance Initiative (COSI 2015–2017) reported a combined overweight/obesity prevalence of 42.5% in boys and 42.1% in girls at age 8 in Spain [3]. Maternal pre-pregnancy obesity is associated with higher odds of childhood obesity in systematic evidence from human observational studies [4]. Beyond immediate maternal risks such as pre-eclampsia and gestational diabetes, maternal adiposity is associated with offspring adiposity and later obesity, although observational associations do not by themselves establish intrauterine causation [5,6,7]. Potential mechanisms can be considered within the exposome framework, which encompasses nongenetic exposures from conception onward [8]. Environmental obesogens may affect adipocyte development and endocrine regulation, illustrating one potential pathway through which early exposures could influence later obesity [9].

Pregnancy is characterised by progressive insulin resistance [10] and changes in maternal fatty acid availability and placental transfer that support foetal growth [11]. Within this context, maternal nutrition is a modifiable influence on foetal development and later metabolic health [12]. Long-chain polyunsaturated fatty acids (LC-PUFAs) have well-established roles in brain and retinal development [13,14]; they serve as structural membrane components and as ligands for transcription factors that regulate adipogenesis and inflammation [15]. Because the foetus depends almost entirely on maternal and placental supply, LC-PUFAs are delivered across the placenta via specific fatty acid transport and binding proteins, a process that becomes increasingly important as gestation advances [16,17,18,19]. Adipocytes continue to turn over during adulthood [20], while early developmental windows may influence later adiposity [21]. Mechanistically, adipogenesis involves the differentiation of mesenchymal stem cells into mature adipocytes, a process tightly regulated by transcription factors such as peroxisome proliferator-activated receptor gamma (PPAR-γ) and CCAAT/enhancer-binding protein α (C/EBP-α) [22]. Fatty acids can regulate gene transcription through direct and indirect mechanisms [23]. Genetic variation can further modulate these pathways: the Pro12Ala polymorphism in the PPAR-γ-2 gene, for instance, has been associated with lower BMI and improved insulin sensitivity [24].

Maternal obesity and accompanying metabolic disturbances are associated with offspring adiposity and later cardiometabolic disease [5,6,7]. Desaturases contribute to regulation of fatty acid composition [25], and dietary intervention studies have examined desaturase indices in different lipid compartments [26]. These pathways may contribute to variation in fatty-acid profiles during pregnancy. Altered maternal lipid metabolism and placental fatty acid handling are potential pathways through which maternal metabolic status may influence foetal development [19]. This dysregulation is of particular relevance in obese pregnancies, where it may compound foetal lipid exposure.

Evidence from animal models suggests that maternal obesity or high-fat feeding can impair placental function and alter offspring adipose-tissue development [21,27]. Maternal obesity has been associated with altered expression and secretion of angiogenic and adhesion molecules in maternal omental adipose tissue; in addition, maternal obesity exacerbates these effects by disrupting placental lipid transfer and amplifying inflammatory signalling [28]; pro-inflammatory cytokines derived from maternal adipose tissue, together with altered placental mediator production, contribute to reduced insulin sensitivity [29] and may modify the intrauterine lipid environment and foetal exposure to bioactive fatty acids, with consequences for long-term metabolic programming [30]. Maternal nutritional status and eating behaviour before and during pregnancy have been investigated in relation to offspring obesity, with much of the mechanistic evidence derived from animal models [30]. Linoleic acid (LA) is a precursor of arachidonic acid (ARA), which can be metabolised to bioactive eicosanoids involved in inflammation and adipocyte differentiation; dietary LA intake alone does not establish the extent of this conversion in pregnancy [31]. Epidemiological studies further suggest an inverted U-shaped association between maternal LA intake and birth weight, with both low and high intakes linked to reduced birth weight [32], and excessive LA exposure has additionally been associated with increased maternal inflammation, altered placental lipid composition, and impaired foetal growth. In a dietary intervention study, *n*-3 supplementation and counselling to reduce ARA intake lowered maternal and cord-blood *n*-6/*n*-3 LC-PUFA ratios, but the maternal ratio was not linked to early postnatal adipose-tissue development [33]. Postnatal fatty acid exposure may also vary with maternal weight status: a PREOBE follow-up reported higher breast-milk *n*-6/*n*-3 ratios and lower ALA and DHA in women with overweight or obesity [34], which may alter adipose-tissue development and increase offspring susceptibility to obesity later in life.

Historical estimates suggest that Western diets contain substantially more *n*-6 than *n*-3 PUFAs [35], and US food-availability data document marked increases in LA consumption during the twentieth century [36]. Maternal fatty acid requirements during pregnancy and lactation must be considered in relation to foetal and infant supply [37]. Mechanistic evidence, mainly from rodent models, indicates that *n*-3 PUFAs can reduce adipose-tissue inflammation and influence thermogenesis [38]; these findings do not establish that changing maternal PUFA intake prevents childhood obesity. The relevance of altered maternal PUFA profiles to offspring adiposity in pregnancies affected by overweight or obesity (OWO) remains uncertain. A systematic review of pregnancy supplementation trials and observational studies reported inconsistent observational findings and a small increase in childhood BMI after higher-dose DHA and/or EPA supplementation, underscoring uncertainty about long-term effects on adiposity [39].

Here, we investigated associations between maternal PUFA status during pregnancy and in cord blood and offspring adiposity and body composition at 8.5 years of age across maternal normal-weight, overweight, and obese groups.

## 2. Materials and Methods

### 2.1. Ethics, Informed Consent and Permissions

The PREOBE study was conducted in accordance with the updated principles of the Declaration of Helsinki [40,41]. The project was approved by the Provincial Ethics Committees for Clinical Research (CEI) of Granada (1 June 2016-GD-BRAIN and and 13 May 2026-PREOBE FOLLOW-UP-DYNAHEALTH), and previously at the Ethics Research Committee of the University of Granada (13 July 2006-PREOBE Excellence Project). All families were informed about the study protocol, and written informed consent was obtained from each parent or legal guardian before their child was enrolled.

### 2.2. Study Design and Subjects

The present study is based on data collected within the PREOBE study (www.ClinicalTrials.gov, identifier NCT01634464), the details of which have been published elsewhere [42]. Briefly, 331 pregnant women were recruited between 2008 and 2012 at the San Cecilio Clinical University Hospital, the Mother-Infant University Hospital of Granada, and affiliated medical centres, and were followed up until delivery (n = 310). In the original cohort, 269 women were recruited before 24 weeks and 62 after 24 weeks of gestation. Inclusion criteria were a singleton pregnancy between 12 and 34 weeks of gestation (preferably before 20 weeks); maternal age between 18 and 45 years; no concurrent enrolment in another research project; no drug treatment; no vegan diet; and no pre-existing conditions other than overweight, obesity, or gestational diabetes mellitus. Offspring were followed up with until 8.5 years of age.

**Figure 1:**
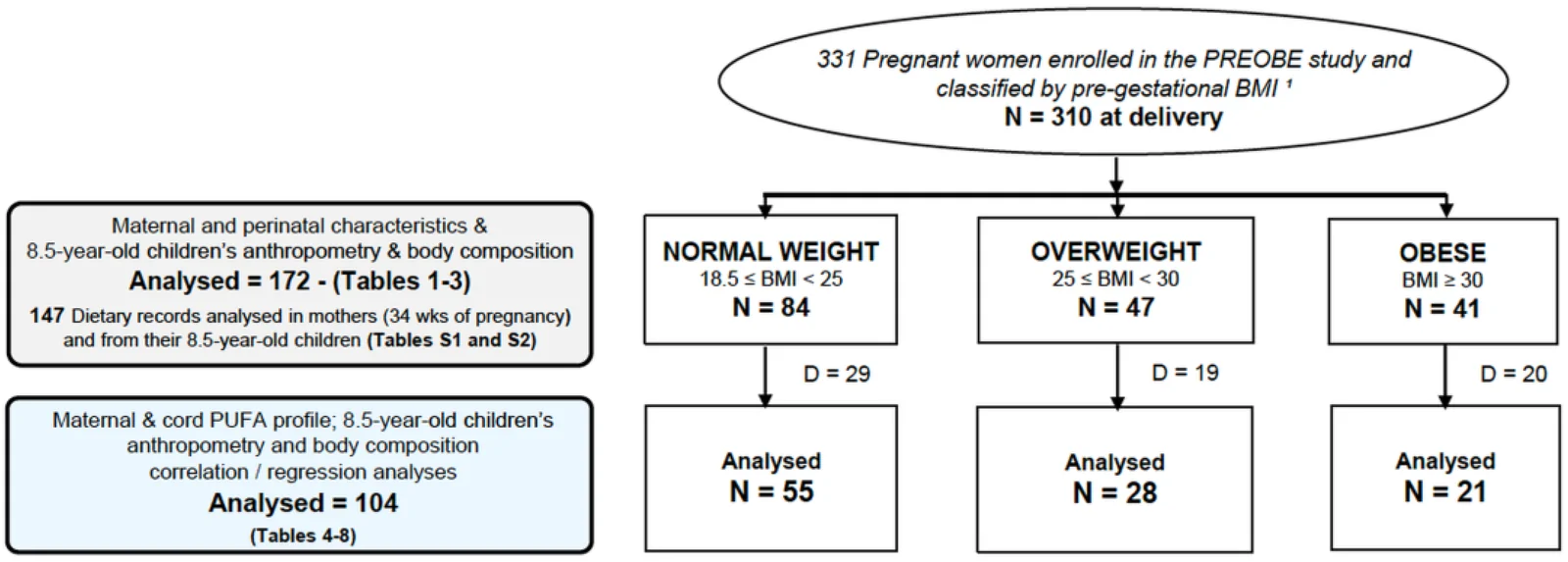
Participant flow diagram for the present study (PREOBE cohort). ^1^ The n = 172 mother–child pairs (normal weight, overweight, obese) analysed here are drawn from the wider PREOBE cohort. Baseline maternal/child characteristics, sociodemographics and 8.5-year anthropometric and body-composition data are reported for the full n = 172. In addition, 147 dietary records obtained in 8.5-year-old children and their mothers at 34 weeks of pregnancy were analysed (73 NW, 40 OW, 34 OB). Analyses of maternal and cord-blood plasma phospholipid PUFA profiles and their correlation/regression with offspring anthropometry and body composition are restricted to the n = 104 mother–child pairs (NW = 55, OW = 28, OB = 21) with a complete fatty-acid profile at all four blood-sampling timepoints and valid 8.5-year anthropometry and body-composition data. D = dropouts; reasons for missing data: incomplete plasma phospholipid PUFA profile at ≥1 gestational/cord timepoint (refused blood draws, missed contact at delivery, and the narrow window for cord-blood collection) and/or missing 8.5-year body-composition data due to loss of contact, unwillingness to attend the visit, or relocation to another city (n = 68 excluded in total).

In the present study, a total of 172 children were categorised into three groups based on their mothers’ pregestational body mass index (BMI): normal weight (NW): 18.5 ≤ BMI < 25 (n = 84), overweight (OW): 25 ≤ BMI < 30 (n = 47) and obese (OB): BMI ≥ 30 (n = 41). The n = 172 mother–child pairs analysed here are drawn from the wider PREOBE cohort (Figure 1). Baseline maternal/child characteristics, sociodemographics, and 8.5-year anthropometric and body-composition data are presented for this descriptive cohort. The number of available observations varies by measure. In addition, 147 dietary records obtained in 8.5-year-old children and their mothers at 34 weeks of pregnancy were analysed (NW = 73, OW = 40, OB = 34). Analyses of maternal and cord-blood plasma phospholipid PUFA profiles and their correlation/regression with offspring anthropometry and body composition are restricted to the n = 104 mother–child pairs (NW = 55, OW = 28, OB = 21) with a complete fatty-acid profile at all four blood-sampling timepoints and valid 8.5-year anthropometry and body-composition data. Reasons for missing data were incomplete plasma phospholipid PUFA profile at ≥1 gestational/cord timepoint (refused blood draws, missed contact at delivery, and the narrow window for cord-blood collection) and/or missing 8.5-year body-composition data due to loss of contact, unwillingness to attend the visit, or relocation to another city (n = 68 excluded in total).

### 2.3. Demographical and Clinical Characteristics

At the recruitment visit, data were collected on maternal age, pre-pregnancy BMI, parity, sociodemographic and medical background, ethnicity, education, smoking habits, and alcohol consumption; pregnant women were also asked about current or prior use of vitamin or mineral supplements. Maternal venous blood samples were collected by arm venipuncture at follow-up visits at 24 and 34 weeks of gestation. Participants were additionally instructed to contact the study centre upon admission to the delivery ward, where maternal venous blood was collected at delivery (approximately 40 weeks of gestation) together with umbilical cord blood, obtained immediately after cord clamping. Mode of delivery and placental weight were also recorded. Medical records were reviewed to determine maternal weight at delivery and to calculate gestational weight gain (GWG). After delivery, neonatal data were extracted from clinical records, including sex, gestational age, and neonatal anthropometry (birth weight, birth length, head circumference).

At the children’s follow-up visit at 8.5 years of age, data were collected on socioeconomic status, parental smoking, physical activity level using the validated FELS Physical Activity Questionnaire [43], and children’s current medication and chronic disease status.

### 2.4. Children’s Anthropometry Assessment at 8.5 Years Old

Children’s anthropometric data, including weight, height, BMI, mid-upper arm circumference (MUAC), waist circumference (WC), and tricipital and subscapular skinfolds (TSF and SSF), were obtained by a trained nutritionist at 8.5 years of age, following standard procedures of the World Health Organization (WHO) and the International Society for the Advancement of Kinanthropometry (ISAK) [44,45]. Weight was measured with a TANITA body-composition analyser BC-418MA (Biologica TM S.L., Barcelona, Spain); height with a SECA 264 stadiometer (max. 220 cm); waist circumference with a SECA measuring tape; and skinfolds with a Holtain Model DIM-98610ND calliper (Holtain Ltd, Croswell, Wales, UK) (max. 40 mm). Weight-for-age, height-for-age, and BMI-for-age z-scores were calculated using the WHO growth reference for school-aged children [44]. Waist-to-height ratio (WHtR) was calculated as waist circumference (cm) divided by height (cm).

• Body fat mass

Body fat mass (BFM) was estimated using two complementary methodologies: anthropometric skinfold equations and bioelectrical impedance analysis with TANITA^®^.

• Anthropometric skinfolds measurement

BFM percentage was calculated from the TSF and SSF using Slaughter’s equations [46] as follows, where ΣSF is the sum of the TSF and SSF (mm):

When ΣSF was ≤35 mm, the equations used were

- BFM (%) = 1.21 × ΣSF − 0.008 × (ΣSF)^2^ − 1.7 for boys;
- BFM (%) = 1.33 × ΣSF − 0.013 × (ΣSF)^2^ − 2.5 for girls.

When ΣSF was >35 mm, the equations used were

- BFM (%) = 0.783 × ΣSF + 1.6 for boys;
- BFM (%) = 0.546 × ΣSF + 9.7 for girls.

Fat-free mass (FFM) percentage was calculated as 100 − FM%. Fat mass (kg) and FFM (kg) were obtained by multiplying body weight (kg) by the corresponding percentage divided by 100. Lastly, the fat mass index [FMI (kg/m^2^) = FM (kg)/height (m)^2^] and the fat-free mass index [FFMI (kg/m^2^) = FFM (kg)/height (m)^2^] were calculated.

• Bioelectrical impedance

Body composition was also assessed using a Tanita BC-418MA^®^ Body Composition Analyzer, which indirectly estimates total body water, FM, and FFM using an electrical current (50 kHz, 90 µA) delivered via eight electrodes. This method is based on the principle that body water conductivity varies across body compartments [47,48].

### 2.5. Children’s and Maternal Dietary Intake

Maternal dietary intake at 34 weeks of gestation was recorded for at least three days using a seven-day dietary record form (Supplementary Table S1) [49]. Children’s dietary intake at 8.5 years of age was assessed using three-day dietary records. DIAL 1.0 software (Alce Ingeniería, Madrid, Spain) [50] was used to convert reported food consumption into macronutrient and micronutrient intakes (Supplementary Table S2).

### 2.6. Fatty Acid Analysis

Maternal and umbilical cord-blood samples collected during pregnancy and at delivery were used to determine the fatty-acid (FA) profile of plasma phospholipids (PL). Plasma was separated by centrifugation, immediately frozen, and stored at −80 °C until analysis. Fatty acids were quantified in the phospholipid fraction as previously described by Chisaguano et al. [51]: plasma lipids were extracted using a 2:1 dichloromethane:methanol solution, and phospholipids were isolated by solid-phase extraction (SPE). Fatty acid methyl esters derived from plasma phospholipids were analysed by gas chromatography with flame ionisation detection (FID), and results were expressed as a percentage of total fatty acids detected.

### 2.7. Statistical Analysis

All statistical analyses were performed using R version 4.1.2. Normality of continuous variables was assessed using the Shapiro–Wilk test; descriptive values are reported as means and standard errors. ANOVA, Bonferroni post hoc comparisons, and ANCOVA were used to compare study groups, adjusting by gestational weight gain, DHA supplementation during pregnancy, maternal education level, and household income and child medication at 8.5 years. Categorical variables were summarised as frequencies and percentages and compared using chi-square tests. Maternal plasma phospholipid fatty acid percentages exhibited non-normal distributions on Shapiro–Wilk testing, which informed the choice of bivariate Spearman rank correlations between fatty acids and offspring anthropometry/body composition within each maternal BMI group. Nominal raw *p*-values and Benjamini–Hochberg False Discovery Rate (FDR)-adjusted q-values are reported separately in Supplementary Table S3 and Table S4. FDR correction addresses multiple testing, rather than confounding; q < 0.05 was used to identify FDR-supported associations. FDR correction was applied separately within each maternal BMI group and within each fatty acid family, across the correlations reported (19 *n*-3 and 43 *n*-6 correlations; the correlation analyses were therefore considered exploratory. Robust linear regression analyses used the pooled sample of 104 mother–child pairs for Models 2 and 3; Model 1 corresponds to the unadjusted comparison in all children with available outcome data (n = 172). Model 1 included maternal pregestational BMI category, with normal weight as the reference. Model 2 additionally included maternal age, gestational weight gain, maternal education, and household income. Model 3 added maternal and cord-blood fatty acids selected stepwise. Adjustment in these pooled models does not confer covariate adjustment on the subgroup correlations. Nominal *p* < 0.05 was the significance threshold for other analyses.

No a priori sample size calculation was performed for this secondary analysis, as the sample was determined by data availability in the PREOBE cohort. A sensitivity power analysis (two-sided α = 0.05; power = 80%) indicated that the minimum detectable Spearman correlation (Fisher z-transformation with variance 1.06/(n − 3)) was |ρ| = 0.38 in the NW group (n = 55), 0.52 in the OW group (n = 28), and 0.59 in the OB group (n = 21). For the three-group comparisons, the minimum detectable effect size was Cohen’s f = 0.24 (n = 172) and f = 0.31 (n = 104), and for a single predictor in the pooled regression models (n = 104) it was f^2^ = 0.08.

In addition, an exploratory classification-tree analysis was performed using recursive partitioning (rpart package in R) [52] to investigate potential nonlinear and hierarchical associations between maternal, anthropometric, and fatty acid variables and childhood BMI category according to the US Preventive Services Task Force classification (normal weight, overweight, and obesity). The analysis was intended to identify candidate predictors and data-derived threshold values rather than to develop or validate a clinical prediction rule. The classification tree was generated using recursive binary partitioning, displaying the number of participants and the BMI-category proportions for each terminal node. Because the split points were determined empirically from the study sample, they were considered exploratory thresholds and were not interpreted as clinically validated cut-offs. Given the sample size and the number of candidate variables, together with incomplete observations for several biochemical measures, the possibility of model overfitting was considered. Accordingly, the decision-tree findings were considered exploratory and hypothesis-generating. No external validation cohort was available, and therefore the generalizability of the identified split points and terminal-node distributions could not be established.

## 3. Results

Maternal pre-conceptional BMI, GWG, DHA supplementation, and educational level differed across the three study groups (Table 1). There were no statistical differences in the incidence of gestational diabetes mellitus (GDM) between the study groups. Only four obese mothers (9.75%) reported taking DHA supplements during pregnancy, compared with thirty-seven (90.25%) who did not. In contrast, 29.76% of normal-weight mothers reported taking DHA supplements during pregnancy. Most obese mothers had a primary or secondary level of education (n = 32; 78%), whereas nine (n = 9; 21.95%) had a university-level education (Table 1).

At 34 weeks of pregnancy, OB mothers reported lower energy (kcal/day) and carbohydrates intake and higher LC-PUFAs *n*-6/*n*-3 intake compared to NW and OW mothers. No differences between groups were observed regarding the intake of other macronutrients or other FAs (Supplementary Table S1; n = 147: NW = 73, OW = 40, OB = 34).

At 8.5 years, household income and medication use differed across maternal BMI groups (overall *p* = 0.027 and *p* = 0.003, respectively; Table 2). The proportion of households with annual income below €18,000 was highest in the OB group, and medication use was most frequent in the OW group. No differences were found depending on chronic diseases and physical activity level of children, family status and parental smoking habits. Assessment of the 3-day food record collected by parents (Supplementary Table S2; n = 147: NW = 73, OW = 40, OB = 34) showed that children from the OB group consumed less kcal/day, carbohydrates, sugar and saturated fatty acids compared with those born to OW and NW mothers. Intake of other macronutrients and FAs was homogeneous between groups.

### 3.1. Children’s Body Composition at 8.5 Years Old Depending on Their Mothers’ Pre-Pregnancy BMI

Children born to OB mothers had higher weight, weight-for-age and BMI-for-age z-scores, MUAC and its z-score, waist circumference and its z-score, waist-to-height ratio (WHtR), and triceps skinfold thickness and its z-score than those born to NW mothers (Table 3). Height, height-for-age z-score, and FFMI did not differ significantly *amonggroups*. The comparisons in Table 3 were adjusted for GWG, maternal DHA supplementation, maternal education, household income, and children’s medication use at 8.5 years. No significant pairwise differences were observed between the OW and OB groups.

Children born to OB mothers also had higher skinfold-derived FM percentage, FM (kg), FMI, and FMI/FFMI ratio, and lower FFM percentage than children born to NW mothers. Bioimpedance measurements showed higher FM percentage, total fat mass, trunk fat mass (percentage and kg), total FFM (kg), and total body water (kg) in the OB group than in the NW group (Table 3).

### 3.2. Maternal and Umbilical Cord FA Levels in Plasma PL During Pregnancy Depending on Pregestational BMI

As shown in Table 4, there were some significant differences during pregnancy in the levels of *n*-3 PUFAs in plasma PL depending on maternal pregestational BMI; alpha-linolenic acid (ALA) percentages were lower in OWO mothers at 24 and 40 weeks of gestation, compared with NW mothers. DHA percentages in plasma PL were only different at 34 weeks, being lower in OWO pregnant women.

At 34 weeks of gestation, adrenic acid (AdA), total *n*-6 PUFAs, *n*-6 LC-PUFAs, and the total and long-chain *n*-6/*n*-3 ratios differed across maternal BMI groups. The post hoc comparisons showed higher total *n*-6 PUFA percentages and LC-PUFA *n*-6/*n*-3 ratios in both OW and OB mothers than in NW mothers. For *n*-6 LC-PUFAs and the total PUFA *n*-6/*n*-3 ratio, the indicated pairwise difference was between OB and NW mothers, with the OW group intermediate. AdA differed overall at 34 weeks (adjusted *p* = 0.039), with the highest mean in the OB group. At 40 weeks, *n*-6 LC-PUFAs also differed across groups (adjusted *p* = 0.019). Cord-blood fatty acid percentages did not differ significantly across groups (Table 5).

### 3.3. Correlation Analysis of Maternal Fatty Acids During Pregnancy and Offspring’s Body Composition at 8.5 Years of Age

Maternal *n*-3 PUFA correlations with childhood anthropometry and body composition are reported in Table 6, with raw *p*-values and FDR-adjusted q-values in Supplementary Table S3. In the OW group, ALA at 34 weeks was positively correlated with BAZ, MUACZ, TSF, TSFZ, skinfold-derived FM percentage, FMI, FMI/FFMI, and bioimpedance trunk FM percentage, and inversely correlated with bioimpedance total FFM (kg); all these associations had q < 0.05. DPA at 34 weeks was positively correlated with trunk FM percentage (q = 0.008). At 40 weeks, ALA and DPA were inversely correlated with HAZ; ALA was inversely correlated with skinfold-derived FFM (kg) and bioimpedance FM percentage but positively correlated with bioimpedance total FFM (kg), whereas DPA was inversely correlated with bioimpedance total FFM (kg) (all q < 0.05). In the NW group, no association at 34 weeks met the FDR threshold. At 40 weeks, ALA was positively correlated with HAZ and skinfold-derived FFM (kg), and DPA with WAZ, HAZ, and bioimpedance total FFM (kg) (all q < 0.05). In the OB group, ALA at 40 weeks and DPA at 34 weeks were positively correlated with FFMI at the nominal level (*p* = 0.019 and *p* = 0.024, respectively), but neither association survived FDR correction (both q = 0.228). No OB-group association in Supplementary Table S3 met q < 0.05.

Maternal and cord-blood *n*-6 PUFA correlations with childhood body composition are reported in Table 7, with nominal *p*-values and FDR-adjusted q-values in Supplementary Table S4. The following descriptions distinguish nominal associations from those meeting q < 0.05. Differences in significance across groups do not establish differences in association strength; no formal between-group comparison of correlations is reported.

#### 3.3.1. Normal-Weight Mothers (NW)

At 24 weeks, maternal LA was inversely correlated with skinfold-derived FM percentage and FFMI at the nominal level, but these associations did not survive FDR correction in Supplementary Table S4 (q = 0.065 and q = 0.056, respectively). At 34 weeks, positive associations of LA with MUACZ, EDA with MUAC and MUACZ, and ARA with WAZ met the FDR threshold. The inverse GLA–MUACZ association did not (q = 0.054). At 40 weeks, GLA was inversely correlated with HAZ (q = 0.039), while its association with height did not meet the FDR threshold (q = 0.054). Cord GLA was inversely correlated with weight, WAZ, waist circumference, WCZ, WHtR, BAZ, TSFZ, skinfold- and bioimpedance-derived FM percentage and kg, FFMI, FMI/FFMI, and trunk FM percentage (all q < 0.05). Cord GLA was also inversely correlated with skinfold- and bioimpedance-derived FFM percentage and kg (all q < 0.05). Cord AdA was nominally positively correlated with FMI/FFMI, but this did not survive FDR correction (q = 0.054).

#### 3.3.2. Overweight Mothers (OW)

At 24 weeks, maternal LA was positively correlated with TSF, TSFZ, skinfold-derived FM percentage, FMI, and FFMI (all q = 0.036). The positive AdA–bioimpedance FFM (kg) and inverse AdA–FMI/FFMI associations were nominal only (q = 0.059 and q = 0.063, respectively). At 34 weeks, ARA was inversely correlated with bioimpedance FFM percentage and kg, and AdA with bioimpedance FFM (kg) (all q < 0.05). No association at 40 weeks met the FDR threshold. Cord GLA was inversely correlated with waist circumference, WCZ, skinfold-derived FM (kg), FFMI, FMI/FFMI, bioimpedance FFM (kg), and trunk FM percentage (all q < 0.05). Its associations with BAZ and TSFZ were nominal only (q = 0.063 and q = 0.053), and the WHtR association was not nominally significant (*p* = 0.060, q = 0.099). Cord ARA was positively correlated with BAZ and FMI/FFMI; cord DGLA was inversely correlated with bioimpedance FM (kg); and cord AdA was inversely correlated with BAZ and positively correlated with bioimpedance FFM (kg) (all q < 0.05). The inverse cord AdA–FMI/FFMI association did not survive FDR correction (q = 0.079).

#### 3.3.3. Obese Mothers (OB)

No OB-group *n*-6 PUFA association in Supplementary Table S4 met q < 0.05. At 40 weeks, the PUFA *n*-6/*n*-3 ratio was nominally positively correlated with FMI/FFMI (*p* = 0.046), but this did not survive FDR correction (q = 0.989). Cord GLA was nominally inversely correlated with FFMI (ρ = −0.52, *p* = 0.016, q = 0.688). The cord ARA–FFMI association was not statistically significant (ρ = −0.28, *p* = 0.219, q = 1.000). These results do not establish a lower-fat-free-mass phenotype or the absence of an association in the population.

Pooled robust linear regression results for 104 mother–child pairs (Models 2 and 3; Model 1 included all 172 children) are presented in Table 8. Maternal BMI category coefficients compare the OW and OB groups with the NW reference group; the fatty acid coefficients describe pooled associations and are not estimates specific to NW mothers.

### 3.4. Pooled Fatty Acid Associations

In Model 3, cord GLA was inversely associated with BAZ, WCZ, WHtR, body fat mass percentage, and FMI (all reported *p* ≤ 0.012). These are covariate-adjusted associations within the pooled model, rather than evidence of independent causal effects.

In contrast, maternal GLA levels at 40 weeks of gestation were positively associated with BFM percentage and FMI (*p* = 0.014).

#### 3.4.1. Overweight Mothers (OW)

In Model 1, maternal overweight was associated with higher BAZ, WCZ, WHtR, body fat mass percentage, and FMI than the NW reference category (all reported *p* < 0.05). According to the reported *p*-values, these coefficients were no longer nominally significant in Models 2 and 3. Changes across models do not establish mediation or identify the reason for the change in estimates.

The Model 3 coefficients for maternal overweight should therefore be interpreted with their reported uncertainty, rather than as evidence that GLA explains the group differences.

#### 3.4.2. Obese Mothers (OB)

In Model 1, maternal obesity was associated with higher BAZ, WCZ, WHtR, body fat mass percentage, and FMI than the NW reference category (all reported *p* < 0.001). According to the reported *p*-values, the maternal obesity coefficients were no longer nominally significant in Models 2 and 3.

The opposite directions of the maternal and cord GLA coefficients in Model 3 concern body fat mass percentage and FMI. Maternal GLA at 40 weeks was not retained for BAZ, WCZ, or WHtR. No group-specific GLA coefficients or formal BMI-by-GLA interaction tests are reported in Table 8.

**Figure 2:**
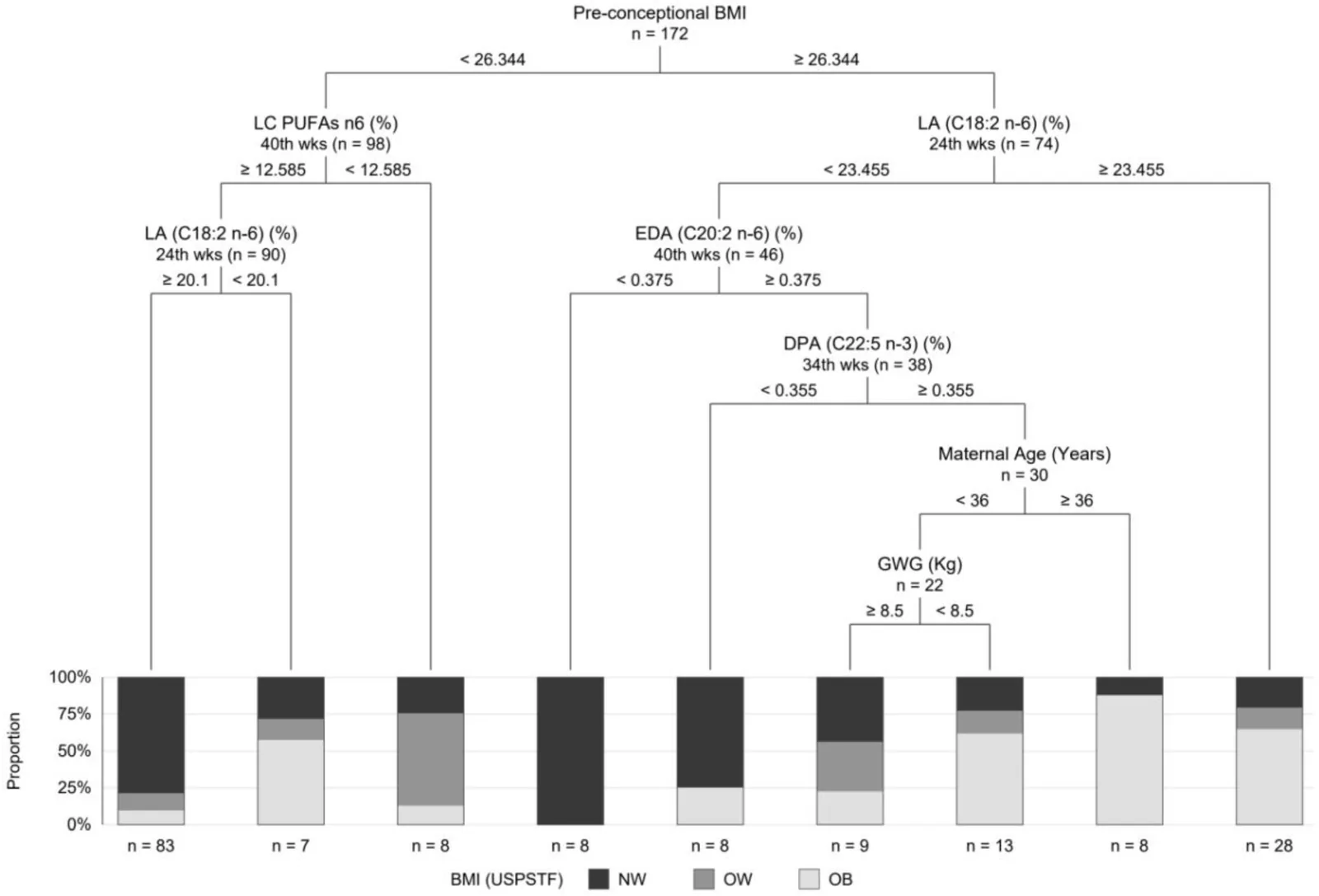
Exploratory classification tree of children’s BMI category at 8.5 years old. The tree illustrated hierarchical associations between different prenatal and perinatal factors together with maternal and umbilical cord long-polyunsaturated fatty acids (LC-PUFAs) during pregnancy and the children’s BMI category according to the US Preventive Services Task Force (USPSTF) classification. Values at each terminal node indicate the number of participants and the observed distribution of normal weight (NW), overweight (OW), and obesity (OB). Split points were derived empirically from the study population and are presented as exploratory thresholds rather than clinically validated cut-offs. Terminal-node proportions should not be interpreted as individual risk estimates. The analysis was exploratory and hypothesis-generating.

An exploratory classification tree examined hierarchical associations between maternal characteristics, plasma phospholipid fatty acids, and children’s BMI category at 8.5 years (Figure 2). The tree included 172 participants. Maternal pre-conceptional BMI was the first split (<26.344 kg/m^2^, n = 98; ≥26.344 kg/m^2^, n = 74), followed by maternal LC-PUFAs *n*-6 at 40 weeks, LA at 24 weeks, *eicosadienoic acid* (EDA) at 40 weeks, *docosapentaenoic acid (DPA)* at 34 weeks, maternal age, and gestational weight gain (GWG) (Kg).

On the maternal BMI ≥ 26.344 kg/m^2^ branch, most children in the LA ≥ 23.455% node (n = 28) were classified as obese. Among children with LA < 23.455% at 24 weeks, EDA ≥ 0.375% at 40 weeks, DPA ≥ 0.355% at 34 weeks, and maternal age ≥ 36 years, the terminal node (n = 8) also had a high observed proportion of obesity. In contrast, all eight children in the EDA < 0.375% node were classified as normal weight. On the maternal BMI < 26.344 kg/m^2^ branch, most children in the node defined by LC-PUFAs *n*-6 ≥ 12.585% at 40 weeks and LA < 20.1% at 24 weeks (n = 7) were classified as obese, whereas those in the LC-PUFAs *n*-6 < 12.585% node (n = 8) were mostly overweight. These displayed proportions describe small, exploratory subgroups and are not individual risk estimates.

## 4. Discussion

The present study examined associations of maternal pregestational BMI and serial maternal and cord-blood phospholipid fatty-acid profiles with childhood body composition at 8.5 years against the background of a high prevalence of childhood obesity [3]. The repeated measurements describe variation across gestation and between maternal and cord compartments, but do not directly identify mechanisms of developmental programming.

The observed PUFA associations can be considered within integrative exposome frameworks linking maternal adiposity, diet, and metabolic exposures with offspring health [53]. The reported *p*-values for maternal BMI category coefficients exceeded 0.05 in the adjusted regression models. This change does not establish mediation by fatty acids or show that maternal BMI is merely a proxy for a modifiable metabolic exposure. Group-stratified correlations remained unadjusted for covariates, and their interpretation differs from that of the pooled regression models.

### 4.1. Maternal Pregestational BMI and Offspring Body-Composition Phenotype

Consistent with prior epidemiological and mechanistic evidence, maternal pregestational BMI was strongly associated with offspring adiposity at 8.5 years across several metrics, including higher weight, BMI-for-age z-score, waist circumference, triceps skinfold, fat mass (FM) and fat mass index (FMI), alongside a reduced fat-free mass percentage (FFM%), but not height. These findings describe differences in adiposity without corresponding differences in height [54]. Higher trunk fat and waist-to-height ratio (WHtR) in the OB group indicate greater central adiposity; children of OB mothers also displayed a higher FMI/FFMI ratio, indicating more fat mass relative to fat-free mass, which includes tissues other than skeletal muscle [54]. Associations between adiposity and *n*-3 fatty acid status have also been reported in adults [55]. A recent UK birth-cohort study associated maternal and paternal pre-pregnancy adiposity with metabolic dysfunction-associated steatotic liver disease (MASLD) in offspring at 24 years, with childhood BMI contributing to the observed association [56]. The single childhood body-composition assessment does not establish an adiposity-rebound trajectory or demonstrate an intrauterine causal effect.

### 4.2. Gestational Remodelling of the Maternal Fatty-Acid Profile in Overweight and Obesity

LC-PUFA accumulation accelerates during the third trimester, and lower circulating DHA and ARA levels have previously been reported in preterm compared with term infants [57]. We found no between-group differences in cord ARA or DHA. Maternal ALA percentages were lower in OW and OB mothers than in NW mothers at 24 and 40 weeks, while DHA was lower at 34 weeks (Table 4). At 34 weeks, total *n*-6 PUFA percentages and the LC-PUFA *n*-6/*n*-3 ratio were higher in both OW and OB groups than in NW mothers; for *n*-6 LC-PUFAs and the total PUFA *n*-6/*n*-3 ratio, the indicated pairwise difference was between OB and NW mothers, with OW intermediate (Table 5). AdA differed overall at 34 weeks, with its highest mean in the OB group. These findings can be considered alongside published observations of altered maternal lipid metabolism in obesity [58,59,60]. These findings raise hypotheses about inflammatory pathways in OWO pregnancies; inflammatory mediators were not assessed in the present analysis [61]. This pattern is consistent with observations in non-pregnant populations: obese women have been shown to display a lower omega-3 index and lower erythrocyte *n*-3 PUFA proportions than normal-weight women independent of dietary *n*-3 intake, suggesting altered fat absorption, utilisation, or storage rather than diet alone [62]. Several studies in non-pregnant populations have reported associations between adiposity and *n*-3 fatty acid status [55,63,64,65,66]. Studies in adults have examined fish-oil intake in relation to fat oxidation [67], adipokine-mediated metabolic changes in obesity [68], and changes in erythrocyte membranes after weight loss [69]. Oxidative stress is another proposed pathway in foetal programming [70]. These findings provide context, but do not identify the cause of the plasma PUFA differences observed here.

### 4.3. Maternal and Foetal n-3 PUFAs and Body Composition

The *n*-3 PUFA associations varied descriptively by maternal BMI group and gestational timepoint. In the OW group, maternal ALA at 34 weeks was positively associated with several adiposity measures, whereas ALA at delivery was positively associated with skinfold-derived FFM in the NW group. In the OB group, the nominal positive correlations of ALA at delivery and DPA at 34 weeks with FFMI did not survive FDR correction (both q = 0.228), and do not support a conclusion that these fatty acids preserve lean mass [54]. Evidence outside pregnancy indicates that fatty-acid profiles vary with metabolic status: serum ALA and DHA were inversely associated with abdominal obesity in a cross-sectional study of 60-year-old adults [71]. A small dietary weight-loss study in adults with obesity also found increased *n*-3 LC-PUFA incorporation into skeletal-muscle phospholipids [72]. Early nutritional exposures may influence later metabolism through epigenetic regulation [73], but these studies do not establish that higher maternal DPA preserves offspring lean mass. In a randomised trial, 2.4 g/day of *n*-3 LC-PUFA from 24 weeks of gestation to one week postpartum was associated with higher offspring BMI and odds of overweight at 10 years, without statistically significant differences in fat or lean mass [74]. These findings caution against inferring a childhood adiposity benefit from mechanistic or observational associations alone.

The *n*-6 PUFA findings also varied by sampling time and compartment. In the NW group, cord GLA was inversely correlated with several adiposity measures, but also with FFM measures; therefore, these data do not support a general claim of improved lean mass. In the OW group, maternal LA at 24 weeks was positively associated with adiposity measures, while several inverse cord GLA–adiposity associations met the FDR threshold. These descriptive patterns do not establish that the associations differ between maternal BMI groups.

Cord GLA showed several inverse unadjusted correlations with adiposity measures in the NW and OW groups. In the pooled regression model, it was inversely associated with adiposity outcomes after adjustment for the listed covariates. These are distinct analyses; neither establishes a protective effect or a BMI-dependent response. In prepubertal children, plasma fatty-acid profiles differed with obesity and metabolic syndrome, with lower ARA proportions associated with metabolic syndrome [75]. In rats, GLA-rich borage-oil diets were associated with less fat accumulation and greater brown-adipose UCP1 expression than a linoleic-acid-rich safflower-oil diet [76]. GLA is elongated to DGLA, a precursor of prostaglandin E1; however, elevated DGLA also occurs in obesity, so its concentration cannot be interpreted as uniformly anti-inflammatory [77]. Recent lipidomic work has reported statistical mediation by specific cord-blood lipid species for part of the association between maternal BMI and birth weight [78]. This provides a rationale for further biomarker research, without establishing causal mediation in the present study. Cord-blood fatty acid composition integrates both maternal nutrient supply and placental transfer efficiency, and placental transport selectively regulates LC-PUFA delivery to the foetus [17]; maternal obesity is, in turn, consistently linked to altered placental function, low-grade inflammation, insulin resistance, and dysregulated lipid metabolism, which provide potential biological pathways for further investigation [5,6].

This is consistent with recent evidence from the same PREOBE cohort showing that only maternal dietary DHA intake in the third trimester remained significantly associated with the corresponding placental phospholipid percentage after consideration of potential confounders. Modest unadjusted correlations were also observed for myristic, stearic, oleic, and α-linolenic acids, whereas placental ARA was not correlated with reported dietary ARA intake [79]. These findings suggest that relationships between reported diet and placental fatty acids may differ by fatty acid: GLA and ARA, which depend heavily on maternal desaturase activity for their generation, may reflect both dietary and metabolic influences, consistent with the mechanistic model proposed above.

The maternal and cord GLA coefficients had opposite directions in the pooled models for body fat mass percentage and FMI: higher cord GLA wasassociated with lower values, whereas higher maternal GLA at 40 weeks was associated with higher values. Cord GLA was also inversely associated with BAZ, WCZ, and WHtR, but maternal GLA at 40 weeks was not retained for these outcomes. Differences in metabolic processing and placental transport are possible explanations; however, the study did not measure GLA conversion flux, demonstrate a metabolic blockade, or establish effective foetal utilisation from a cord-blood percentage. The findings therefore warrant mechanistic investigation rather than a causal interpretation. Transcriptional and epigenetic regulation of PPARγ provides a general mechanistic context for adipocyte differentiation [80].

Several mechanisms could be investigated to explain differences between maternal and cord fatty-acid profiles. LA is converted to GLA by Δ6-desaturase (FADS2), GLA is elongated to DGLA by elongases, and DGLA is converted to ARA by Δ5-desaturase (FADS1). Associations of estimated desaturase activities with obesity and metabolic risk provide a rationale for examining these pathways [81]. Placental fatty acid transporters, including FATP2, FATP4, and CD36, may also contribute to variation in foetal fatty acid supply [82]. Obesity-related inflammation, insulin resistance, and oxidative stress are further candidate influences on placental lipid handling [70]. These mechanisms were not directly measured here, and the present data do not establish an obesity-related disruption of GLA transfer. A nonlinear or threshold relationship remains a hypothesis; no GLA threshold was tested or validated. Placental expression studies, enzyme-activity measurements, and formal interaction and nonlinear analyses would be required to evaluate these possibilities.

Within the OB group, the cord GLA–FFMI correlation was nominally significant (ρ = −0.52, *p* = 0.016), but not after FDR correction (q = 0.688). The cord ARA–FFMI correlation was not significant (ρ = −0.28, *p* = 0.219), and the maternal *n*-6/*n*-3 ratio–FMI/FFMI association at 40 weeks did not survive FDR correction (q = 0.989). Although experimental and observational studies have examined *n*-6 exposure, adiposity, and desaturase activity [58,83,84], these OB-group results do not establish a distinct body-composition phenotype. Previous paediatric work has considered sarcopenic obesity and cardiovascular risk [85], but FFMI is not a specific measure of skeletal muscle mass, and muscle function was not assessed in this study. The consensus framework requires assessment of muscle function and body composition [86]; accordingly, neither sarcopenic obesity nor a sarcopenic-obesity-like phenotype can be inferred from these correlations.

### 4.4. Potential Mechanisms Linking PUFAs and Adipose Development

Several biological pathways could contribute to the reported associations. LC-PUFAs are structural membrane components and precursors of bioactive lipid mediators, and influence gene expression [87]. PPARγ is a central regulator of adipocyte differentiation whose expression is controlled by transcriptional and epigenetic mechanisms [80]. Rat and mouse experiments have linked maternal fatty-acid balance with offspring adipose-tissue development or metabolism [88,89]; these experimental results do not directly establish a causal mechanism in humans. Reviews of maternal lipid metabolism provide a broader context for these pathways [90], while oxidative stress may also contribute to developmental programming [70].

### 4.5. Dietary Intake Patterns and the Likelihood of Under-Reporting

Plasma fatty-acid profiles reflect dietary intake and endogenous metabolism. Longitudinal work has described changes in circulating lipids during pregnancy [91], and a previous PREOBE cord-blood metabolomic study examined profiles in relation to maternal BMI and GDM [92]. In the present report, dietary records at 34 weeks did not indicate higher absolute *n*-6 intake in OB mothers, despite differences in their plasma phospholipid profiles. This does not establish dietary under-reporting or inflammation. DHA supplementation was reported by 9.75% of OB mothers (Table 1). Published pregnancy dietary guidance provides a context for nutritional assessment [93], but the present analysis did not test adherence to a DHA target or demonstrate a benefit of supplementation. Differences between dietary records and blood fatty acid percentages should therefore be interpreted with caution.

At 8.5 years, children of OB mothers had lower reported carbohydrate and sugar intake than children of NW or OW mothers. Dietary intake assessment in children is vulnerable to reporting error, which can vary with age, body-weight status, and assessment method [94]. A Japanese national survey associated childhood obesity with energy-intake under-reporting [95]. In a controlled study of adult women, omitted between-meal snacks contributed to under-reporting of energy, carbohydrate, and added-sugar intake [96]. These studies establish plausible sources of reporting error, but do not show which participants under-reported in PREOBE or quantify the error in the present records. Plasma phospholipid profiles alone cannot resolve reporting bias or classify overall diet quality.

### 4.6. Socioeconomic Disadvantage and the Intergenerational Obesity Cycle

Maternal obesity in the PREOBE cohort was closely linked to educational and economic disadvantage; mothers with obesity were far more likely to have only primary or secondary education (78%) than NW mothers, of whom 58.3% held university degrees, a gap mirrored by the low rate of DHA supplementation in the OB group (under 10%) [54]. Two Italian cohorts found associations between pregestational BMI and maternal mid-pregnancy erythrocyte PUFA profiles in non-obese pregnancies [97]. Across seven birth cohorts in six high-income countries, lower maternal education and household income in early childhood were associated with higher obesity risk at ages 8–11 years [98]. The potential contribution of maternal *n*-6 and *n*-3 PUFA balance to foetal programming requires further experimental and human study [99]. OB mothers reported lower total energy intake and a higher dietary *n*-6/*n*-3 ratio at 34 weeks. These observations do not establish a pro-inflammatory diet or explain intergenerational obesity; socioeconomic influences warrant consideration [100]. As previously reported, the PREOBE cohort collected maternal and family characteristics; in the present sample, educational level differed by maternal BMI group and family income differed at the 8.5-year follow-up (*p* = 0.027) [42]. Lower socioeconomic status is associated with greater psychological distress, poorer access to healthy food and active lifestyles, and lower quality of life [100]. In the contemporary Spanish PASOS study, lower socioeconomic status and parental educational level were associated with obesity, severe obesity, and abdominal obesity [100]; historical Spanish survey data also showed socioeconomic inequalities in overweight and obesity trends between 1987 and 2007 [101]. Mean gestational weight gain was higher in OW mothers (10.54 ± 1.06 kg) than in OB mothers (5.32 ± 1.36 kg; overall *p* < 0.001, with a significant post hoc group comparison); group means alone do not establish the proportion of women exceeding Institute of Medicine (IOM) recommendations. Previous meta-analysis has related GWG outside recommended ranges to adverse pregnancy outcomes [102]. At 8.5 years, chronic disease frequency—including type 1 diabetes, allergies, asthma, and coeliac disease—did not differ by maternal pre-conceptional BMI, but reported use of medication, including corticosteroids, NSAIDs, or antibiotics, was most frequent in children of OW mothers (overall *p* = 0.003), while previous work has examined childhood obesity and regular medication use [103].

### 4.7. Exploratory Classification Tree and Comparison with Other Cohorts

The exploratory classification tree identified maternal BMI, LA at 24 weeks, EDA at 40 weeks, DPA at 34 weeks, maternal age, and GWG as splitting variables, with some small terminal nodes containing predominantly children classified as obese. Experimental work has examined maternal PUFA balance and offspring adipose regulation [104]. In the observational Generation R study, maternal PUFA profiles in mid-pregnancy were associated with childhood total and abdominal adiposity [84]. In Project Viva, higher cord DHA was associated with lower BMI z-score in early childhood, but these associations did not persist into mid-childhood [105]. An earlier Project Viva analysis reported inverse associations of maternal dietary DHA plus EPA intake and cord plasma DHA plus EPA with adiposity at age 3, whereas maternal plasma DHA plus EPA was not significantly associated with adiposity [106]. Differences in sampling times, fatty acid measurements, childhood ages, and analytical approaches limit direct comparisons with the present findings.

The literature on prenatal fatty acid exposure and childhood adiposity includes both observational studies and intervention trials, whose findings should be distinguished [58]. Maternal age was a downstream splitting variable in this exploratory tree, together with fatty acid measurements and GWG. Other predictive-modelling studies have also considered maternal age among childhood obesity predictors [107], but such comparisons do not validate the split points identified here. Maternal demographic and nutritional factors may relate to offspring adiposity through several pathways [84]. Our tree did not select an *n*-6/*n*-3 ratio, formally test interactions, or establish that changing a fatty acid value would alter a child’s outcome.

Nevertheless, these results should be taken with caution, because the purpose of this analysis in the present study was to identify potential nonlinear and hierarchical associations and to generate candidate threshold values for further investigation, rather than as a validated prediction model. In fact, terminal nodes varied considerably in size, and therefore the observed proportions should be interpreted cautiously. For example, some terminal nodes contained fewer than 10 participants. Figure 2 presents the BMI-category proportions and corresponding sample size for each terminal node. Importantly, the split points identified by the classification tree were derived from the observed data and should not be interpreted as definitive clinical thresholds. Likewise, the proportion classified as obese within a terminal node describes that subgroup and should not be interpreted as an estimate of individual risk. The classification-tree analysis was therefore considered exploratory and hypothesis-generating, with the identified variables and threshold values requiring confirmation in independent cohorts.

### 4.8. Strengths and Limitations

The main strengths of this study are its prospective longitudinal design, repeated maternal plasma phospholipid PUFA measurements from mid-gestation to delivery, cord-blood measurements, and childhood anthropometry and body-composition assessment at 8.5 years. These features permit examination of associations across sampling times and compartments. ANCOVA and pooled regression models included the covariates specified for each analysis; the subgroup Spearman correlations were unadjusted. The use of both skinfolds and bioimpedance provides complementary estimates of body composition, although the direction and significance of some associations differed by measurement method. These features are relevant to studies of prenatal nutrition and later metabolic health [16].

Some limitations should also be acknowledged. The observational design does not establish causality, and residual confounding remains possible. Group-stratified correlations were not adjusted for maternal or child covariates, and FDR correction does not address this limitation. Small subgroup samples, particularly in the OB group (n = 21 for fatty-acid analyses), limit precision. According to the sensitivity power analysis, only correlations of approximately |ρ| ≥ 0.52 in the OW group and |ρ| ≥ 0.59 in the OB group could be detected with 80% power; nonsignificant results in these groups, particularly in the OB group, should therefore not be interpreted as evidence of no association. A significant association in one group and a nonsignificant association in another does not demonstrate effect modification. Incomplete fatty-acid profiles and missing childhood assessments reduced the analytical sample from 172 to 104 mother–child pairs and may introduce selection bias. Incomplete biochemical measurements add uncertainty to the classification-tree findings. Stepwise selection and exploratory subgroup and tree analyses also warrant cautious interpretation. The number of statistical comparisons was large, and FDR correction was applied within the families of reported correlations rather than across all fatty acid–outcome combinations examined; some associations may therefore be false positives and require replication in larger, independent cohorts. Future studies should consider additional exposures, including environmental toxicants [108]. Dietary under-reporting is possible, but was not quantified. Fatty acid structure may also affect lipid handling through pathways such as CD36 [109].

Maternal dietary intake was assessed at 34 weeks of gestation using records covering at least three days; no dietary assessment immediately before delivery or direct maternal diet–blood fatty acid association analysis is presented here. Dietary data were therefore insufficient to distinguish dietary from endogenous metabolic contributions to the observed fatty-acid profiles. Labour-related changes in maternal lipid metabolism have been described in humans [110], and experimental work in animals suggests that maternal obesity can alter peripartum PUFA profiles [111]. Peripartum diet and labour-associated metabolism remain potential influences that this study could not resolve.

Continuous maternal glycaemic biomarkers, such as fasting glucose and HbA1c, were not analysed in this report. The HAPO study related maternal glycaemia to neonatal adiposity [112], providing a rationale for considering glucose metabolism alongside fatty acid status. GDM frequency was reported descriptively, but GDM was not included in the covariate lists for the models reported here; similar group frequencies do not exclude glycaemic confounding. Future analyses should incorporate available glycaemic biomarkers and examine GDM explicitly. This study cannot determine whether prenatal glycaemia explains the higher adiposity despite lower reported childhood energy intake.

## 5. Conclusions

Our findings describe associations between maternal pregestational BMI, maternal and cord-blood PUFA profiles, and childhood body composition within the DOHaD framework [29,30,61,62]. Several inverse cord GLA–adiposity correlations in the NW and OW groups met the FDR threshold, whereas no OB-group correlation did. This difference in significance does not establish a between-group difference. In pooled regression models, cord GLA was inversely associated with adiposity outcomes, while maternal GLA at 40 weeks was positively associated with body fat mass percentage and FMI. These observations do not demonstrate a protective effect or mediation. The classification tree describes exploratory subgroups and requires further validation; its terminal-node distributions are not individual risk estimates.

As an observational study, the present work demonstrates associations rather than causal or protective effects, and the correlational nature of the fatty-acid/body-composition analyses means that residual confounding and false-positive findings remain possible despite covariate adjustment in selected analyses and FDR correction for the correlation tests. We therefore consider it premature to recommend routine individualised LC-PUFA monitoring or supplementation as a clinical intervention on the basis of these findings alone. Instead, our results support the rationale for further mechanistic studies of maternal–foetal PUFA transfer in obesity, and for adequately powered longitudinal and interventional studies to determine whether modifying maternal PUFA, and specifically GLA, status during pregnancy can causally influence offspring body composition, before such monitoring could be considered for prenatal care.

## Figures and Tables

**Table 1:** General characteristics of the PREOBE mother–infant pairs who participated in the present study.

Pregnant women		NW (n = 84)	OW (n = 47)	OB (n = 41)	*p*-Value
Maternal age ^1^ (years)		31.68 ± 0.46	32.60 ± 0.68	31.56 ± 0.80	0.474
Pre-conceptional BMI ^1^ (kg/m^2^)		22.20 ± 0.18 ^a^	27.18 ± 0.19 ^b^	34.30 ± 0.70 ^c^	<0.001
GWG ^1^ (kg)		11.81 ± 0.66 ^a^	10.54 ± 1.06 ^a^	5.32 ± 1.36 ^b^	<0.001
DHA Supplementation ^2^	Yes	25 (29.76)	10 (21.27)	4 (9.75) ^a^	0.046
	No	59 (70.24)	37 (78.73)	37 (90.25) ^b^	
GDM ^2^	Yes	27 (32.14)	16 (34.78)	15 (36.59)	0.876
	No	57 (67.86)	30 (65.22)	26 (63.41)	
Parity ^2^	0	50 (59.52)	26 (55.31)	18 (43.90)	0.256
	≥1	34 (40.48)	21 (44.68)	23 (56.10)	
Family situation ^2^	Single/Separate	2 (2.38)	1 (2.12)	2 (4.90)	0.688
	Living in partnership/Married	82 (97.62)	46 (97.88)	39 (95.10)	
Maternal educational level ^2^	Primary/Secondary	35 (41.66)	27 (57.45)	32 (78.00) ^a^	<0.001
	University	49 (58.33)	20 (42.55)	9 (21.95) ^b^	
Smoking during pregnancy ^2^	Yes	11 (13.25)	6 (12.77)	7 (17.50)	0.779
	No	72 (86.75)	41 (87.23)	33 (82.50)	
Alcohol consumption during pregnancy ^2^	Yes	4 (4.82)	1 (2.12)	2 (5.00)	0.721
	No	79 (95.18)	46 (97.88)	38 (95.00)	
Type of delivery ^2^	Eutocic	52 (62.65)	23 (48.94)	26 (63.41)	0.145
	Dystocic	14 (16.86)	9 (19.15)	2 (4.88)	
	Caesarean	17 (20.48)	15 (31.91)	13 (31.71)	
Placental weight ^1^ (g)		496.22 ± 11.81	504.36 ± 16.69	531.91 ± 20.77	0.331
**Neonate**					
Sex ^2^	Boy	46 (54.76)	23 (48.94)	22 (53.65)	0.809
	Girl	38 (45.24)	24 (51.06)	19 (46.34)	
Gestational age ^1^ (weeks)		39.35 ± 0.13	39.24 ± 0.23	39.63 ± 0.25	0.463
Birth weight ^1^ (g)		3329.76 ± 44.95	3279.15 ± 71.97	3402.17 ± 77.42	0.408
Birth length ^1^ (cm)		50.67 ± 0.21	50.06 ± 0.31	50.72 ± 0.41	0.252
Type of feeding at 3 months ^2^	Breast-feeding	46 (54.76)	23 (48.93)	16 (39.03)	0.238
	Mixed	16 (19.05)	10 (21.27)	6 (14.63)	
	Infant formula	22 (26.20)	14 (29.80)	19 (46.34)	

BMI: body mass index; DHA: docosahexaenoic acid; GWG: gestational weight gain; GDM: gestational diabetes mellitus. Study groups: Children born to mothers with pregestational BMI categorised as NW—normal weight, OW—overweight or OB—obese. ^1^ Values expressed as mean ± standard error of the mean; ^2^ values expressed as number of cases (percentage). Statistical analysis performed: ANOVA: analysis of variance for normally distributed variables. Chi-square test for categorical variables. Values who do not share the same suffix (^a,b,c^) are significantly different in a Bonferroni post hoc test. *p*: Level of significance for overall differences between PREOBE-groups. *p*-value ≤ 0.05 was considered statistically significant.

**Table 2:** Sociodemographic characteristics of the PREOBE children at 8.5 years of age, depending on their mothers’ pregestational BMI.

Children 8.5 Years		NW (n = 84)	OW (n = 47)	OB (n = 41)	*p*-Value
Age (years)		8.56 ± 0.04	8.75 ± 0.05	8.61 ± 0.09	0.754
Family status	Single/Separate	2 (2.43)	3 (6.38)	3 (7.32)	0.395
	Lives in partnership/Married	80 (97.56)	44 (93.62)	38 (92.68)	
Family income (€/year)	<18.000	12 (14.63) ^a^	15 (31.91)	14 (35.00)	0.027
	18.000–33.000	33 (40.24) ^b^	21 (44.68)	14 (35.00)	
	33.000–53.000	27 (32.93)	5 (10.63)	10 (25.00)	
	53.000–300.000	10 (12.20)	6 (12.76)	2 (5.00)	
Smoking (at least one parent)	Yes	31 (36.90)	20 (43.47)	20 (50.00)	0.370
	No	53 (63.10)	26 (56.52)	20 (50.00)	
* Medication	Yes	9 (10.72) ^a^	16 (34.78)	7 (17.08) ^a^	0.003
	No	75 (89.28) ^b^	30 (65.22)	34 (82.92) ^b^	
** Chronic Disease	Yes	26 (30.95)	15 (32.61)	16 (39.02)	0.663
	No	58 (69.05)	31 (67.39)	25 (60.98)	

Study Groups: Children born to mothers with pregestational BMI categorised as NW—normal weight, OW—overweight or OB—obese. * Medication: corticosteroids, non-steroidal anti-inflammatories, antibiotics. ** Chronic diseases: type 1 diabetes mellitus, allergies, asthma, celiac disease. Number of cases (percentage) for categorical variables. Statistical analysis performed: Chi-square test for categorical variables. Values who do not share the same suffix (^a,b^) are significantly different in a Bonferroni post hoc test. *p*: Level of significance for overall differences between the study groups. *p*-value ≤ 0.05 was considered statistically significant.

**Table 3:** Children’s anthropometric measurements and body composition at 8.5 years, classified depending on their maternal pre-conceptional BMI.

Anthropometry	NW Group (n = 84)	OW Group (n = 47)	OB Group (n = 41)	*p* ^1^	*p*-adj.
Weight (kg)	31.06 ± 0.79 ^a^	34.59 ± 1.23 ^a,b^	36.61 ± 1.47 ^b^	0.001	0.003
WAZ	0.61 ± 0.13 ^a^	1.03 ± 0.19 ^b^	1.40 ± 0.22 ^b^	0.004	0.006
Height (cm)	132.73 ± 0.65	133.81 ± 0.91	134.08 ± 0.83	0.396	0.259
HAZ	0.28 ± 0.11	0.26 ± 0.13	0.39 ± 0.13	0.789	0.559
BAZ	0.60 ± 0.13 ^a^	1.13 ± 0.21 ^a,b^	1.55 ± 0.23 ^b^	0.001	0.003
MUAC (cm)	20.04 ± 0.30 ^a^	21.54 ± 0.50 ^b^	22.42 ± 0.58 ^b^	<0.001	0.001
MUACZ	0.14 ± 0.12 ^a^	0.64 ± 0.18 ^b^	0.93 ± 0.19 ^b^	0.001	0.004
WC (cm)	59.55 ± 0.69 ^a^	63.07 ± 1.21 ^b^	65.34 ± 1.49 ^b^	<0.001	0.001
WCZ	0.07 ± 0.09 ^a^	0.48 ± 0.13 ^a,b^	0.70 ± 0.16 ^b^	0.001	0.003
WHtR	0.45 ± 0.01 ^a^	0.47 ± 0.01 ^a,b^	0.49 ± 0.01 ^b^	<0.001	0.002
TSF (mm)	13.76 ± 0.63 ^a^	16.99 ± 1.07 ^b^	18.59 ± 1.21 ^b^	<0.001	0.003
TSFZ	0.70 ± 0.10 ^a^	1.10 ± 0.17 ^a,b^	1.36 ± 0.16 ^b^	0.002	0.011
FM (%) ^1^	19.41 ± 0.67 ^a^	22.86 ± 1.23 ^a,b^	25.95 ± 1.51 ^b^	<0.001	<0.001
FM (kg) ^1^	6.35 ± 0.42 ^a^	8.49 ± 0.76 ^a,b^	10.23 ± 0.96 ^b^	<0.001	0.001
FFM (%) ^1^	80.59 ± 0.67 ^a^	77.14 ± 1.23 ^a,b^	74.05 ± 1.51 ^b^	<0.001	<0.001
FFM (kg) ^1^	24.71 ± 0.43	26.10 ± 0.58	26.38 ± 0.70	0.049	0.038
FMI	3.60 ± 0.21 ^a^	4.76 ± 0.41 ^a,b^	5.57 ± 0.49 ^b^	<0.001	<0.001
FFMI	14.18 ± 0.23	14.76 ± 0.31	14.63 ± 0.31	0.254	0.439
FMI/FFMI ratio	0.25 ± 0.01 ^a^	0.31 ± 0.02 ^a,b^	0.38 ± 0.03 ^b^	<0.001	<0.001
**Bioimpedance ^2^**					
FM (%) ^2^	22.35 ± 0.59 ^a^	25.27 ± 0.98 ^a,b^	27.67 ± 1.26 ^b^	<0.001	<0.001
TFM (kg) ^2^	7.25 ± 0.42 ^a^	9.04 ± 0.71 ^a,b^	10.87 ± 0.96 ^b^	0.001	0.002
TFFM (kg) ^2^	23.84 ± 0.42 ^a^	25.03 ± 0.61 ^a,b^	26.05 ± 0.65 ^b^	0.015	0.011
Trunk FM (%) ^2^	16.30 ± 0.58 ^a^	19.59 ± 1.02 ^a,b^	21.66 ± 1.23 ^b^	<0.001	<0.001
Trunk FM (kg) ^2^	3.01 ± 0.18 ^a^	3.96 ± 0.34 ^a,b^	4.57 ± 0.41 ^b^	<0.001	<0.001
TBW (kg) ^2^	17.45 ± 0.31 ^a^	18.1 ± 0.49 ^a,b^	19.08 ± 0.47 ^b^	0.022	0.032

BAZ: BMI-for-age z-score; BMI: body mass index; FFM: fat-free mass; FFMI: fat-free mass index; FM: fat mass, FMI: fat mass index; HAZ: height-for-age z-score; MUAC: mid-upper-arm circumference; MUACZ: mid-upper-arm-for-age z-score; TBW: total body water; TFM: total fat mass; TFFM: total fat-free mass; TSF: triceps skinfold; TSFZ: tricipital skinfold z-score; WAZ: weight-for-age z-score; WC: waist circumference; WCZ: waist circumference-for-age z-score; WHtR: waist-to-height ratio. Study groups: Children born to mothers with pregestational BMI categorised as NW—normal weight, OW—overweight, OB—obese. Fat mass and fat-free mass calculated by ^1^ Slaughter Equation and ^2^ Bioimpedance (TANITA). Variables are expressed as mean ± standard error of the mean. SEMS reported as 0.00 are <0.005 due to rounding. Statistical analysis performed: ANOVA: analysis of variance for normally distributed variables. Values who do not share the same suffix (^a,b^) are significantly different after post hoc Bonferroni test. *p*-value: Level of significance for overall differences between study groups. *p*-value ≤ 0.05 was considered statistically significant. ANCOVA, *p*-adj.: *p*-value adjusted by gestational weight gain, DHA supplementation during pregnancy, maternal education level, and household income and child medication at 8.5 years.

**Table 4:** Maternal omega 3 polyunsaturated fatty acids (*n*-3 PUFAs) in plasma phospholipids at 24th, 34th, 40th gestational week and in umbilical cord.

	24 Weeks					34 Weeks				
Omega 3	NW	OW	OB	*p*	*p*-adj.	NW	OW	OB	*p*	*p*-adj.
ALA (18:3*n*-3)	0.14 ± 0.01 ^a^	0.12 ± 0.01 ^b^	0.10 ± 0.00 ^b^	<0.001	0.005	0.13 ± 0.01	0.11 ± 0.01	0.10 ± 0.00	0.031	0.078
EPA (20:5*n*-3)	0.44 ± 0.04	0.33 ± 0.04	0.30 ± 0.03	0.154	0.282	0.36 ± 0.03	0.33 ± 0.04	0.34 ± 0.05	0.840	0.822
DPA (22:5*n*-3)	0.44 ± 0.01 ^a^	0.41 ± 0.02 ^a^	0.37 ± 0.01 ^b^	0.049	0.177	0.40 ± 0.01	0.36 ± 0.01	0.37 ± 0.02	0.190	0.197
DHA (22:6*n*-3)	4.75 ± 0.13	4.53 ± 0.16	4.25 ± 0.15	0.214	0.371	4.88 ± 0.12 ^a^	4.39 ± 0.14 ^b^	4.35 ± 0.10 ^b^	0.042	0.045
PUFAs *n*-3	5.77 ± 0.16	5.38 ± 0.20	5.02 ± 0.18	0.100	0.218	5.77 ± 0.14	5.19 ± 0.18	5.17 ± 0.21	0.066	0.081
LC-PUFAs *n*-3	5.63 ± 0.16	5.26 ± 0.20	4.92 ± 0.18	0.126	0.256	5.64 ± 0.14	5.07 ± 0.18	5.07 ± 0.21	0.078	0.093
	**40 Weeks**					**Umbilical Cord**				
**Omega 3**	**NW**	**OW**	**OB**	***p***	***p*** **-adj.**	**NW**	**OW**	**OB**	***p***	***p*** **-adj.**
ALA (18:3*n*-3)	0.13 ± 0.01 ^a^	0.10 ± 0.01 ^b^	0.09 ± 0.01 ^b^	0.001	0.010	0.04 ± 0.00	0.04 ± 0.00	0.03 ± 0.00	0.516	0.647
EPA (20:5*n*-3)	0.34 ± 0.02	0.35 ± 0.04	0.28 ± 0.02	0.368	0.689	0.16 ± 0.02	0.17 ± 0.01	0.15 ± 0.01	0.113	0.307
DPA (22:5*n*-3)	0.40 ± 0.01	0.37 ± 0.01	0.38 ± 0.02	0.556	0.651	0.43 ± 0.02	0.42 ± 0.02	0.35 ± 0.03	0.541	0.637
DHA (22:6*n*-3)	4.59 ± 0.12	4.35 ± 0.15	4.39 ± 0.17	0.564	0.384	6.84 ± 0.20	6.64 ± 0.14	6.79 ± 0.32	0.927	0.501
PUFAs *n*-3	5.46 ± 0.13	5.18 ± 0.18	5.14 ± 0.20	0.491	0.470	7.57 ± 0.22	7.26 ± 0.16	7.32 ± 0.36	0.840	0.548
LC-PUFAs *n*-3	5.33 ± 0.13	5.08 ± 0.18	5.05 ± 0.20	0.574	0.516	7.53 ± 0.22	7.23 ± 0.16	7.29 ± 0.36	0.844	0.547

Study groups: children born to mothers with pregestational BMI categorised as NW—normal weight (n = 55), OW—overweight (n = 28) or OB—obese (n = 21). ALA (18:3*n*-3): α-linolenic acid; DHA (22:6*n*-3): docosahexaenoic acid; DPA (22:5*n*-3): docosapentaenoic acid; EPA (20:5*n*-3): eicosapentaenoic acid; LC-PUFAs: long-chain polyunsaturated fatty acids; PUFAs: polyunsaturated fatty acids. Fatty acids are expressed as percentages of the total fatty acids in plasma phospholipids. Variable data are expressed as mean ± standard error of the mean. SEMS reported as 0.00 are <0.005 due to rounding. ANOVA: Analysis of variance for normally distributed variables was performed. Values who do not share the same suffix (^a,b^) are significantly different after post hoc Bonferroni test. *p*-value: Level of significance for overall differences between study groups. *p*-value ≤ 0.05 was considered statistically significant. ANCOVA, *p*-adj.: *p*-value adjusted by gestational weight gain, DHA supplementation during pregnancy, maternal education level, and household income and child medication at 8.5 years.

**Table 5:** Maternal omega 6 polyunsaturated fatty acids (*n*-6 PUFAs) in plasma phospholipids at 24th, 34th, 40th gestational week and in umbilical cord.

	*24 Weeks*					*34 Weeks*				
*Omega 6*	NW	OW	OB	*p*	*p*-adj.	NW	OW	OB	*p*	*p*-adj.
LA (18:2*n*-6)	23.24 ± 0.27	22.96 ± 0.33	23.43 ± 0.38	0.784	0.703	23.11 ± 0.27	23.44 ± 0.36	23.62 ± 0.41	0.682	0.766
GLA (18:3*n*-6)	0.05 ± 0.00	0.06 ± 0.00	0.05 ± 0.00	0.242	0.298	0.04 ± 0.00	0.05 ± 0.00	0.05 ± 0.00	0.369	0.630
EDA (20:2*n*-6)	0.48 ± 0.01	0.45 ± 0.01	0.45 ± 0.02	0.263	0.432	0.43 ± 0.01	0.40 ± 0.01	0.42 ± 0.02	0.386	0.218
DGLA (20:3*n*-6)	3.66 ± 0.10	3.77 ± 0.13	4.07 ± 0.14	0.205	0.220	3.47 ± 0.08	3.58 ± 0.09	3.76 ± 0.12	0.284	0.103
ARA (20:4*n*-6)	10.13 ± 0.10	10.11 ± 0.11	10.10 ± 0.10	0.169	0.173	9.27 ± 0.18	10.03 ± 0.27	10.28 ± 0.28	0.031	0.094
AdA (22:4*n*-6)	0.36 ± 0.03	0.33 ± 0.04	0.34 ± 0.05	0.171	0.218	0.33 ± 0.01	0.38 ± 0.02	0.39 ± 0.02	0.020	0.039
PUFAs *n*-6	38.05 ± 0.25	38.51 ± 0.28	39.29 ± 0.28	0.076	0.127	37.08 ± 0.27 ^a^	38.29 ± 0.24 ^b^	38.95 ± 0.32 ^b^	0.002	0.006
LC-PUFAs *n*-6	14.76 ± 0.22	15.50 ± 0.23	15.81 ± 0.28	0.057	0.050	13.97 ± 0.20 ^a^	14.85 ± 0.26 ^a,b^	15.33 ± 0.28 ^b^	0.008	0.017
PUFAs *n*-6/*n*-3	7.12 ± 0.24	7.72 ± 0.34	8.23 ± 0.29	0.128	0.314	6.80 ± 0.19 ^a^	7.83 ± 0.31 ^b^	7.97 ± 0.29 ^a,b^	0.012	0.015
LC-PUFAs *n*-6/*n*-3	2.83 ± 0.10	3.16 ± 0.13	3.38 ± 0.14	0.042	0.102	2.61 ± 0.08 ^a^	3.06 ± 0.10 ^b^	3.18 ± 0.11 ^b^	0.001	0.002
	***40 Weeks***					***Cord Blood***				
***Omega 6***	**NW**	**OW**	**OB**	***p***	***p*** **-adj.**	**NW**	**OW**	**OB**	***p*** **-value**	***p*** **-adj.**
LA (18:2*n*-6)	22.53 ± 0.27	21.98 ± 0.39	22.42 ± 0.51	0.695	0.855	8.47 ± 0.25	7.45 ± 0.2	7.35 ± 0.24	0.142	0.310
GLA (18:3*n*-6)	0.05 ± 0.00	0.05 ± 0.00	0.05 ± 0.00	0.774	0.740	0.09 ± 0.00	0.08 ± 0.00	0.08 ± 0.00	0.647	0.552
EDA (20:2*n*-6)	0.43 ± 0.01	0.41 ± 0.01	0.44 ± 0.02	0.643	0.474	0.42 ± 0.01	0.43 ± 0.01	0.38 ± 0.02	0.334	0.138
DGLA (20:3*n*-6)	3.77 ± 0.08	3.95 ± 0.09	4.05 ± 0.10	0.245	0.079	5.70 ± 0.12	5.52 ± 0.17	5.71 ± 0.21	0.859	0.929
ARA (20:4*n*-6)	9.36 ± 0.19	9.83 ± 0.26	10.27 ± 0.27	0.127	0.086	18.30 ± 0.22	19.02 ± 0.24	19.08 ± 0.31	0.335	0.624
AdA (22:4*n*-6)	0.38 ± 0.01	0.39 ± 0.01	0.41 ± 0.01	0.604	0.580	0.88 ± 0.02	0.96 ± 0.03	0.84 ± 0.03	0.378	0.410
PUFAs *n*-6	37.07 ± 0.24	37.17 ± 0.28	38.15 ± 0.32	0.151	0.240	34.83 ± 0.27	34.56 ± 0.38	34.50 ± 0.39	0.903	0.620
LC-PUFAs *n*-6	14.49 ± 0.24	15.13 ± 0.24	15.68 ± 0.31	0.047	0.019	26.28 ± 0.22	27.02 ± 0.25	27.07 ± 0.29	0.314	0.350
PUFAs *n*-6/*n*-3	7.18 ± 0.21	7.62 ± 0.29	7.96 ± 0.37	0.312	0.451	4.93 ± 0.15	4.87 ± 0.13	5.40 ± 0.40	0.666	0.791
LC-PUFAs *n*-6/*n*-3	2.86 ± 0.08	3.11 ± 0.10	3.27 ± 0.12	0.082	0.167	3.73 ± 0.11	3.83 ± 0.10	4.23 ± 0.29	0.481	0.645

Study groups: children born to mothers with pregestational BMI categorised as NW: normal weight (n = 55), OW: overweight (n = 28) or OB: obese (n = 21). ARA (20:4*n*-6): arachidonic acid; AdA (22:4*n*-6): adrenic acid; DGLA (20:3*n*-6): dihomo-γ-linolenic acid; EDA (20:2*n*-6): eicosadienoic acid; GLA (18:3*n*-6): γ-linolenic acid; LA (18:2*n*-6): linoleic acid; PUFAs: polyunsaturated fatty acids; LC-PUFAs: long-chain polyunsaturated fatty acids. Fatty acids are expressed as percentages of the total fatty acids determined in plasma phospholipids. Variable’s data are expressed as mean ± standard error of the mean. SEMS reported as 0.00 are <0.005 due to rounding. ANOVA: Analysis of variance for normally distributed variables was performed. Values who do not share the same suffix (^a,b^) are significantly different after post hoc Bonferroni test. *p*-value: Level of significance for overall differences between study groups. *p*-value ≤ 0.05 was considered statistically significant. ANCOVA, *p*-adj.: *p*-value adjusted by gestational weight gain, DHA supplementation during pregnancy, maternal education level, and household income and child medication at 8.5 years.

**Table 6:** Significant correlations found between maternal *n*-3 long-chain polyunsaturated fatty acids (*n*-3 LC-PUFAs) in plasma phospholipids during pregnancy and anthropometric and body-composition values of their offspring at 8.5 years old.

*n*-3 LC-PUFAs (wks of Gestation)	Children’s Body Composition at 8.5 yrs	NW (n = 55)		OW (n = 28)		OB (n = 21)	
		r	*p*	r	*p*	r	*p*
ALA (C18:3*n*-3) (34w)	BAZ	−0.17	0.215	0.41	0.030 *	−0.12	0.604
	MUACZ	0.18	0.189	0.67	<0.001 *	−0.19	0.410
	TSF	0.04	0.772	0.61	<0.001 *	−0.08	0.730
	TSFZ	−0.16	0.243	0.65	<0.001 *	−0.09	0.698
	FM (%) ^1^	−0.15	0.274	0.60	<0.001 *	−0.11	0.635
	FMI	−0.11	0.424	0.47	0.012 *	−0.13	0.574
	FMI/FFMI	−0.20	0.143	0.51	0.006 *	−0.19	0.410
	TFFM (kg) ^2^	0.22	0.107	−0.52	0.005 *	0.05	0.830
	Trunk FM (%)	−0.03	0.828	0.62	<0.001 *	−0.11	0.635
ALA (C18:3*n*-3) (40w)	HAZ	0.49	<0.001 *	−0.42	0.026 *	−0.39	0.081
	FFM (kg) ^1^	0.41	0.002 *	−0.49	0.008 *	−0.19	0.410
	FFMI	0.02	0.885	0.10	0.613	0.51	0.019
	FM (%) ^2^	0.14	0.308	−0.57	0.002 *	0.23	0.316
	TFFM (kg) ^2^	0.09	0.513	0.48	0.010 *	−0.19	0.410
DPA (C22:5*n*-3) (34w)	FFMI	0.03	0.828	0.12	0.543	0.49	0.024
	Trunk FM (%) ^2^	0.22	0.107	0.54	0.003 *	−0.31	0.171
DPA (C22:5*n*-3) (40w)	WAZ	0.37	0.005 *	0.02	0.920	−0.20	0.385
	HAZ	0.53	<0.001 *	−0.47	0.012 *	−0.40	0.072
	TFFM (kg) ^2^	0.39	0.003 *	−0.52	0.005 *	−0.16	0.488

ALA: α-linolenic acid; BAZ: BMI z-score; DPA: docosapentaenoic acid; FM: fat mass; FMI: fat mass index; FFM: fat-free mass; FFMI: fat-free mass index; HAZ: height-for-age z-score; MUACZ: middle-upper-arm-circumference z-score; TFM: total fat mass; TFFM: total fat-free mass; Trunk FM: trunk fat mass; TSF: triceps skinfold; TSFZ: triceps skinfold-for-age z-score; WAZ: weight-for-age z-score; WCZ: waist-for-age z-score. Study groups: Children born to mothers with pregestational BMI categorised as NW—normal weight, OW—overweight or OB—obese. Fat mass and fat-free mass calculated by ^1^ Slaughter Equation or obtained by ^2^ Bioelectric impedance (TANITA^®^). Fatty acids in maternal plasma phospholipids are expressed as relative percentages of total fatty acids detected. Normality of variables were assessed using Shapiro–Wilk tests. Bivariable Spearman correlation analyses were performed per group. Associations with nominal unadjusted significance (*p* ≤ 0.05) are shown; an asterisk (*) denotes associations remaining statistically significant after Benjamini–Hochberg False Discovery Rate (FDR) correction (q < 0.05). Complete nominal raw *p*-values and FDR-adjusted q-values are provided in Supplementary Table S3.

**Table 7:** Significant correlations found between maternal plasma *n*-6 long-chain polyunsaturated fatty acids in plasma phospholipids during pregnancy and in umbilical cord, and anthropometric and body-composition values of their offspring at 8.5 years old.

		NW (n = 55)		OW (n = 28)		OB (n = 21)	
n-6 LC-PUFAs (Weeks of Gestation)	Children’s Body Composition at 8.5 Years	r	*p*	r	*p*	r	*p*
**24 weeks of pregnancy**							
LA (C18:2*n*-6)	TSF	−0.09	0.513	0.54	0.003 *	0.06	0.796
	TSFZ	−0.23	0.088	0.49	0.008 *	0.02	0.931
	FM (%) ^1^	−0.27	0.042	0.50	0.007 *	−0.01	0.966
	FMI	0.05	0.717	0.50	0.007 *	−0.01	0.966
	FFMI	−0.29	0.031	0.53	0.004 *	−0.04	0.863
AdA (C22:4*n*-6)	TFFM (kg) ^2^	−0.08	0.561	0.41	0.030	−0.06	0.796
	FMI/FFMI	0.09	0.513	−0.40	0.035	0.07	0.763
**34 weeks of pregnancy**							
LA (C18:2*n*-6)	MUACZ	0.31	0.021 *	0.05	0.801	−0.06	0.796
GLA (C18:3*n*-6)	MUACZ	−0.28	0.038	0.08	0.686	0.06	0.796
EDA (C20:2*n*-6)	MUAC (cm)	0.59	<0.001 *	−0.17	0.387	−0.16	0.488
	MUACZ	0.60	<0.001 *	−0.23	0.239	−0.19	0.410
ARA (C20:4*n*-6)	WAZ	0.38	0.004 *	0.03	0.880	0.14	0.545
	BFFM (%) ^2^	−0.17	0.215	−0.47	0.012 *	0.11	0.635
	BFFM (kg) ^2^	−0.19	0.163	−0.48	0.010 *	0.12	0.604
AdA (C22:4*n*-6)	FFM (kg) ^2^	0.15	0.274	−0.51	0.006 *	0.13	0.574
**40 weeks of pregnancy**							
GLA (C18:3*n*-6)	Height (cm)	−0.28	0.038	0.06	0.762	0.09	0.698
	HAZ	−0.31	0.021 *	0.02	0.920	0.09	0.698
PUFAs *n*-6/*n*-3 ratio	FMI/FFMI	0.09	0.513	−0.09	0.649	0.44	0.046
Umbilical cord							
GLA (C18:3*n*-6)	Weight (Kg)	−0.45	<0.001 *	−0.07	0.723	0.10	0.666
	WAZ	−0.47	<0.001 *	−0.09	0.649	0.13	0.574
	WC (cm)	−0.48	<0.001 *	−0.46	0.014 *	−0.04	0.863
	WCZ	−0.50	<0.001 *	−0.45	0.016 *	−0.01	0.966
	WHtR	−0.34	0.011 *	−0.36	0.060	−0.19	0.410
	BAZ	−0.46	<0.001 *	−0.40	0.035	−0.01	0.966
	TSFZ	−0.55	<0.001 *	−0.42	0.026	−0.11	0.635
	FM (%) ^1^	−0.48	<0.001 *	−0.02	0.920	−0.02	0.931
	FM (kg) ^1^	−0.51	<0.001 *	−0.44	0.019 *	−0.03	0.897
	FFM (%) ^1^	−0.38	0.004 *	−0.05	0.801	0.27	0.237
	FFM (kg) ^1^	−0.41	0.001 *	−0.04	0.840	0.05	0.830
	FFMI	−0.31	0.018 *	−0.43	0.022 *	−0.52	0.016
	FMI/FFMI	−0.44	<0.001 *	−0.49	0.008 *	−0.05	0.830
	BFM (%) ^2^	−0.49	<0.001 *	−0.06	0.762	−0.16	0.488
	BFM (kg) ^2^	−0.44	<0.001 *	−0.05	0.801	0.09	0.698
	BFFM (%) ^2^	−0.39	0.003 *	−0.08	0.686	0.08	0.730
	BFFM (kg) ^2^	−0.45	<0.001 *	−0.48	0.010 *	−0.003	0.990
	BTrunkFM (%) ^2^	−0.52	<0.001 *	−0.43	0.022 *	−0.08	0.730
DGLA (C20:3*n*-6)	FM (kg) ^2^	−0.01	0.942	−0.49	0.008 *	0.06	0.796
ARA (C20:4*n*-6)	BAZ	−0.17	0.214	0.55	0.002 *	0.07	0.763
	FFMI	0.07	0.612	−0.08	0.686	−0.28	0.219
	FMI/FFMI	−0.23	0.088	0.47	0.012 *	0.09	0.698
AdA (C22:4*n*-6)	BAZ	0.07	0.612	−0.48	0.010 *	0.06	0.796
	BFFM (kg) ^2^	−0.25	0.062	0.44	0.019 *	0.02	0.931
	FMI/FFMI	0.28	0.038	−0.38	0.046	−0.10	0.666

AdA: adrenic acid; ARA: arachidonic acid; BAZ: BMI-for-age z-score; DGLA: dihomo-γ-linolenic acid; EDA: eicosadienoic acid; FFM: fat-free mass; FMI: fat mass index; FFMI: fat-free mass index; GLA: γ-linolenic acid; LA: linoleic acid; HAZ: length for height-for-age z-score; LC-PUFAs: long-chain polyunsaturated fatty acids; MUAC: mid-upper-arm circumference; MUACZ: mid-upper-arm circumference-for-age z-score; PUFAs: polyunsaturated fatty acids; TFM: total fat mass; TFFM: total fat-free mass; TSF: triceps skinfold; TSFZ: triceps skinfold-for-age z-score; WAZ: weight-for-age z-score; WHR: waist-to-height ratio. Comparison groups: Mothers with a pregestational BMI categorised as normal weight; OW: overweight; OB: obese. Fat mass and fat-free mass calculated by ^1^ Slaughter Equation or obtained by ^2^ Bioelectric impedance (TANITA). Fatty acids in maternal plasma phospholipids are expressed as relative percentages of total fatty acids analysed. Normality of variables were assessed using Shapiro–Wilk tests. Bivariable Spearman correlation analyses were performed per group. Associations with nominal unadjusted significance (*p* ≤ 0. 05) are shown; an asterisk (*) denotes associations remaining statistically significant after Benjamini–Hochberg False Discovery Rate (FDR) correction (q < 0.05). Complete nominal raw *p*-values and FDR-adjusted q-values are provided in Supplementary Table S4.

**Table 8:** Robust linear regression analysis of a priori predictors of children’s body composition at 8.5 years of age (Model 1, n = 172; Models 2 and 3, n = 104).

Outcome Comparison	Model 1 B (CI 95%)	*p*-Value *(Outcome)*	*p*-Value *(Model 1)*	Model 2 B (CI 95%)	*p*-Value *(Outcome)*	*p*-Value *(Model 2)*	Model 3 B (CI 95%)	*p*-Value *(Outcome)*	*p*-Value *(Model 3)*
**BAZ**									
Overweight	0.527 (0.049, 1.005)	0.031	<0.001	0.510 (−0.174, 1.194)	0.142	0.064	−0.482 (−1.922, 0.958)	0.508	0.067
Obese	0.945 (0.445, 1.445)	<0.001		0.824 (−0.370, 2.018)	0.174		−0.104 (−2.553, 2.345)	0.933	
GLA—UC							−1.490 (−2.645, −0.335)	0.012	
**WCZ**									
Overweight	0.410 (0.091, 0.729)	0.012	<0.001	0.258 (−0.184, 0.700)	0.249	0.017	0.030 (−0.677, 0.737)	0.933	0.005
Obese	0.623 (0.288, 0.958)	<0.001		0.204 (−0.570, 0.978)	0.602		0.123 (−1.191, 1.437)	0.853	
GLA—UC							−0.920 (−1.601, −0.239)	0.009	
**WHtR**									
Overweight	0.022 (0.004, 0.040)	0.015	<0.001	0.015 (−0.007, 0.037)	0.178	<0.001	−0.003 (−0.036, 0.031)	0.861	<0.001
Obese	0.041 (0.023, 0.059)	<0.001		0.017 (−0.024, 0.058)	0.415		−0.002 (−0.064, 0.060)	0.952	
GLA—UC							−0.490 (−0.870, −0.110)	0.012	
**BFM (%)**									
Overweight	3.445 (0.678, 6.212)	0.015	<0.001	3.441 (−0.432, 7.314)	0.081	0.008	−1.235 (−6.934, 4.464)	0.668	<0.001
Obese	6.533 (3.622, 9.444)	<0.001		6.014 (−0.733, 12.761)	0.080		3.921 (−6.386, 14.228)	0.452	
GLA—40 wks							4.978 (1.031, 8.925)	0.014	
GLA—UC							−8.440 (−13.940, −2.940)	0.003	
**FMI**									
Overweight	1.157 (0.256, 2.058)	0.012	<0.001	1.100 (−0.151, 2.351)	0.084	0.011	−0.251 (−1.837, 1.335)	0.754	<0.001
Obese	1.966 (1.024, 2.908)	<0.001		1.551 (−0.660, 3.762)	0.167		1.052 (−1.986, 4.090)	0.493	
GLA—40 wks							1.421 (0.294, 2.548)	0.014	
GLA—UC							−2.374 (−3.921, −0.827)	0.003	

BAZ: body mass index (BMI)-for-age z-score; WCZ: waist circumference-for-age z-score; WHtR: waist-to-height ratio; BFM: body fat mass; FMI: fat mass index {fat mass (kg)/height (m^2^)}; UC: umbilical cord; 40 wks: 40 weeks of pregnancy. Model 1: Univariable model evaluating maternal pregestational BMI category (overweight and obesity vs. normal-weight reference). Model 2: Model 1 + maternal age + gestational weight gain (GWG) + maternal education level + family household income (potential collinearity was prevented by using categorical maternal BMI as the exposure without adding continuous BMI; variance inflation factors were verified, VIF < 2.5). Model 3: Model 2 + maternal and cord LC-PUFAs selected stepwise in a robust linear regression model. Cord GLA (and maternal GLA at 40 weeks for BFM and FMI) was the only retained significant fatty acid; β: beta coefficient; CI: 95% confidence intervals. B: unstandardised regression coefficient. Model 1 was estimated in all children with available outcome data (n = 172) and corresponds to the unadjusted group comparison shown in Table 3; Models 2 and 3 were estimated in the 104 mother–child pairs with complete covariate and fatty acid data. *p*-value (outcome): two-sided *p*-value for each coefficient; *p*-value (Model): overall significance of the model.

## Data Availability

The original contributions presented in this study are included in the article/Supplementary Materials. Further inquiries can be directed to the corresponding author .

## Supplementary Materials

The following supporting information can be downloaded at https://www.mdpi.com/article/10.3390/nu18203453/s1. Table S1: Daily intake reported by mothers at 34 weeks of pregnancy, depending on maternal pre-pregestational BMI. Table S2: Children’s dietary intake at 8.5 years old by maternal pre-pregestational BMI group. Table S3: Significant correlations between maternal *n*-3 polyunsaturated fatty acids in plasma phospholipids and offspring anthropometry and body composition at 8.5 years, with nominal *p*-values and FDR-adjusted q-values (corresponding to Table 6). Table S4: Significant correlations between maternal or umbilical cord *n*-6 polyunsaturated fatty acids or fatty acid ratios in plasma phospholipids and offspring anthropometry and body composition at 8.5 years, with nominal *p*-values and FDR-adjusted q-values (corresponding to Table 7).

## Abbreviations

The following abbreviations are used in this manuscript:

AdA	Adrenic acid
ALA	Alpha-linolenic acid
ARA	Arachidonic acid
BAZ	BMI-for-age z-score
BFM	Body fat mass
BMI	Body mass index
C/EBP-α	CCAAT/enhancer-binding protein α-
COSI	Childhood obesity surveillance initiative
COX-2	Cyclooxygenase-2
DHA	Docosahexaenoic acid
DGLA	Dihomo-gamma-linolenic acid
DOHaD	Developmental origins of health and disease
DPA	Docosapentaenoic acid
EDA	Eicosadienoic acid
FAO	Food and Agriculture Organization
FFM	Fat-free mass
FFMI	Fat-free mass index
FID	Flame ionisation detection
FM	Fat mass
FMI	Fat mass index
GDM	Gestational diabetes mellitus
GLA	Gamma-linolenic acid
GWG	Gestational weight gain
HAZ	Height-for-age z-score
IOM	Institute of Medicine
ISAK	International Society for the Advancement of Kinanthropometry
LA	Linoleic acid
LC-PUFA	Long-chain polyunsaturated fatty acid
MASLD	Metabolic dysfunction-associated steatotic liver disease
MUAC	Mid-upper arm circumference
NW	Normal weight
OB	Obese
OW	Overweight
OWO	Overweight and obesity
PL	Phospholipids
PPAR-γ	Peroxisome proliferator-activated receptor gamma
PUFA	Polyunsaturated fatty acid
SPE	Solid-phase extraction
SSF	Subscapular skinfold
TBW	Total body water
TFFM	Total fat-free mass
TFM	Total fat mass
TSF	Tricipital skinfold
TSFZ	Tricipital skinfold z-score
WAZ	Weight-for-age z-score
WC	Waist circumference
WCZ	Waist circumference-for-age z-score
WHO	World Health Organization
WHtR	Waist-to-height ratio

## References

[1] Barker D.J.P. (2004). Developmental Origins of Adult Health and Disease. J. Epidemiol. Community Health.

[2] Gluckman P.D., Hanson M.A., Spencer H.G. (2005). Predictive Adaptive Responses and Human Evolution. Trends Ecol. Evol..

[3] Spinelli A., Buoncristiano M., Nardone P., Starc G., Hejgaard T., Júlíusson P.B., Fismen A., Weghuber D., Milanović S.M., García-Solano M. (2021). Thinness, Overweight, and Obesity in 6- to 9-Year-Old Children from 36 Countries: The World Health Organization European Childhood Obesity Surveillance Initiative—COSI 2015–2017. Obes. Rev..

[4] Heslehurst N., Vieira R., Akhter Z., Bailey H., Slack E., Ngongalah L., Pemu A., Rankin J. (2019). The Association between Maternal Body Mass Index and Child Obesity: A Systematic Review and Meta-Analysis. PLoS Med..

[5] Poston L., Caleyachetty R., Cnattingius S., Corvalán C., Uauy R., Herring S., Gillman M.W. (2016). Preconceptional and Maternal Obesity: Epidemiology and Health Consequences. Lancet Diabetes Endocrinol..

[6] Catalano P.M., Shankar K. (2017). Obesity and Pregnancy: Mechanisms of Short Term and Long Term Adverse Consequences for Mother and Child. BMJ.

[7] Godfrey K.M., Reynolds R.M., Prescott S.L., Nyirenda M., Jaddoe V.W.V., Eriksson J.G., Broekman B.F.P. (2017). Influence of Maternal Obesity on the Long-Term Health of Offspring. Lancet Diabetes Endocrinol..

[8] Vrijheid M. (2014). The Exposome: A New Paradigm to Study the Impact of Environment on Health. Thorax.

[9] Heindel J.J., Blumberg B. (2019). Environmental Obesogens: Mechanisms and Controversies. Annu. Rev. Pharmacol. Toxicol..

[10] Barbour L.A., McCurdy C.E., Hernandez T.L., Kirwan J.P., Catalano P.M., Friedman J.E. (2007). Cellular Mechanisms for Insulin Resistance in Normal Pregnancy and Gestational Diabetes. Diabetes Care.

[11] Mauro A.K., Rengarajan A., Albright C., Boeldt D.S. (2022). Fatty Acids in Normal and Pathological Pregnancies. Mol. Cell. Endocrinol..

[12] Koletzko B., Brands B., Poston L., Godfrey K., Demmelmair H. (2012). Early Nutrition Programming of Long-Term Health. Proc. Nutr. Soc..

[13] Innis S.M. (1991). Essential Fatty Acids in Growth and Development. Prog. Lipid Res..

[14] Lauritzen L., Hansen H.S., Jørgensen M.H., Michaelsen K.F. (2001). The Essentiality of Long Chain n-3 Fatty Acids in Relation to Development and Function of the Brain and Retina. Prog. Lipid Res..

[15] Calder P.C. (2020). N-3 PUFA and Inflammation: From Membrane to Nucleus and From Bench to Bedside. Proc. Nutr. Soc..

[16] Haggarty P. (2010). Fatty Acid Supply to the Human Fetus. Annu. Rev. Nutr..

[17] Gil-Sánchez A., Demmelmair H., Parrilla J.J., Koletzko B., Larqué E. (2011). Mechanisms Involved in the Selective Transfer of Long Chain Polyunsaturated Fatty Acids to the Fetus. Front. Genet..

[18] Duttaroy A.K. (2009). Transport of Fatty Acids Across the Human Placenta: A Review. Prog. Lipid Res..

[19] Duttaroy A.K., Basak S. (2022). Maternal Fatty Acid Metabolism in Pregnancy and Its Consequences in the Feto-Placental Development. Front. Physiol..

[20] Spalding K.L., Arner E., Westermark P.O., Bernard S., Buchholz B.A., Bergmann O., Blomqvist L., Hoffstedt J., Näslund E., Britton T. (2008). Dynamics of Fat Cell Turnover in Humans. Nature.

[21] Scheidl T.B., Brightwell A.L., Easson S.H., Thompson J.A. (2023). Maternal Obesity and Programming of Metabolic Syndrome in the Offspring: Searching for Mechanisms in the Adipocyte Progenitor Pool. BMC Med..

[22] Rosen E.D., Spiegelman B.M. (2006). Adipocytes as Regulators of Energy Balance and Glucose Homeostasis. Nature.

[23] Jump D.B. (2004). Fatty Acid Regulation of Gene Transcription. Crit. Rev. Clin. Lab. Sci..

[24] Deeb S.S., Fajas L., Nemoto M., Pihlajamäki J., Mykkänen L., Kuusisto J., Laakso M., Fujimoto W., Auwerx J. (1998). A Pro12Ala Substitution in PPARgamma2 Associated with Decreased Receptor Activity, Lower Body Mass Index and Improved Insulin Sensitivity. Nat. Genet..

[25] Nakamura M.T., Nara T.Y. (2004). Structure, Function, and Dietary Regulation of Delta6, Delta5, and Delta9 Desaturases. Annu. Rev. Nutr..

[26] Warensjö E., Risérus U., Gustafsson I.B., Mohsen R., Cederholm T., Vessby B. (2008). Effects of Saturated and Unsaturated Fatty Acids on Estimated Desaturase Activities During a Controlled Dietary Intervention. Nutr. Metab. Cardiovasc. Dis..

[27] Desai M., Ross M.G. (2011). Fetal Programming of Adipose Tissue: Effects of Intrauterine Growth Restriction and Maternal Obesity/High-Fat Diet. Semin. Reprod. Med..

[28] Lappas M. (2014). Markers of Endothelial Cell Dysfunction Are Increased in Human Omental Adipose Tissue from Women with Pre-Existing Maternal Obesity and Gestational Diabetes. Metabolism.

[29] Hotamisligil G.S. (2006). Inflammation and Metabolic Disorders. Nature.

[30] Sen S., Carpenter A.H., Hochstadt J., Huddleston J.Y., Kustanovich V., Reynolds A.A., Roberts S. (2012). Nutrition, Weight Gain and Eating Behavior in Pregnancy: A Review of Experimental Evidence for Long-Term Effects on the Risk of Obesity in Offspring. Physiol. Behav..

[31] Calder P.C. (2015). Functional Roles of Fatty Acids and Their Effects on Human Health. JPEN J. Parenter. Enter. Nutr..

[32] Nayyar D., Said J.M., McCarthy H., Hryciw D.H., O’Keefe L., McAinch A.J. (2024). Effect of a High Linoleic Acid Diet on Pregnant Women and Their Offspring. Nutrients.

[33] Much D., Brunner S., Vollhardt C., Schmid D., Sedlmeier E.M., Brüderl M., Heimberg E., Bartke N., Boehm G., Bader B.L. (2013). Effect of Dietary Intervention to Reduce the n-6/n-3 Fatty Acid Ratio on Maternal and Fetal Fatty Acid Profile and Its Relation to Offspring Growth and Body Composition at 1 Year of Age. Eur. J. Clin. Nutr..

[34] de la Garza Puentes A., Martí Alemany A., Chisaguano A.M., Montes Goyanes R., Castellote A.I., Torres-Espínola F.J., García-Valdés L., Escudero-Marín M., Segura M.T., Campoy C. (2019). The Effect of Maternal Obesity on Breast Milk Fatty Acids and Its Association with Infant Growth and Cognition—The PREOBE Follow-Up. Nutrients.

[35] Simopoulos A.P. (2002). The Importance of the Ratio of Omega-6/Omega-3 Essential Fatty Acids. Biomed. Pharmacother..

[36] Blasbalg T.L., Hibbeln J.R., Ramsden C.E., Majchrzak S.F., Rawlings R.R. (2011). Changes in Consumption of Omega-3 and Omega-6 Fatty Acids in the United States During the 20th Century. Am. J. Clin. Nutr..

[37] Brenna J.T., Lapillonne A. (2009). Background Paper on Fat and Fatty Acid Requirements During Pregnancy and Lactation. Ann. Nutr. Metab..

[38] Kalupahana N.S., Goonapienuwala B.L., Moustaid-Moussa N. (2020). Omega-3 Fatty Acids and Adipose Tissue: Inflammation and Browning. Annu. Rev. Nutr..

[39] Ren X., Vilhjálmsdóttir B.L., Rohde J.F., Walker K.C., Runstedt S.E., Lauritzen L., Heitmann B.L., Specht I.O. (2021). Systematic Literature Review and Meta-Analysis of the Relationship Between Polyunsaturated and Trans Fatty Acids During Pregnancy and Offspring Weight Development. Front. Nutr..

[40] The World Medical Association (2008). Declaration of Helsinki: Ethical Principles for Medical Research Involving Human Subjects, 2008 Revision.

[41] World Medical Association (2013). World Medical Association Declaration of Helsinki: Ethical Principles for Medical Research Involving Human Subjects. JAMA.

[42] Berglund S.K., García-Valdés L., Torres-Espinola F.J., Segura M.T., Martínez-Zaldívar C., Aguilar M.J., Agil A., Lorente J.A., Florido J., Padilla C. (2016). Maternal, Fetal and Perinatal Alterations Associated with Obesity, Overweight and Gestational Diabetes: An Observational Cohort Study (PREOBE). BMC Public Health.

[43] Treuth M.S., Hou N., Young D.R., Maynard L.M. (2005). Validity and Reliability of the Fels Physical Activity Questionnaire for Children. Med. Sci. Sports Exerc..

[44] World Health Organization Growth Reference 5–19 Years—Application Tools. https://www.who.int/tools/growth-reference-data-for-5to19-years/application-tools.

[45] Norton K. (2018). Standards for Anthropometry Assessment. Kinanthropometry and Exercise Physiology.

[46] Slaughter M.H., Lohman T.G., Boileau R.A., Horswill C.A., Stillman R.J., Van Loan M.D., Bemben D.A. (1988). Skinfold Equations for Estimation of Body Fatness in Children and Youth. Hum. Biol..

[47] Costa Moreira O., Alonso-Aubin D.A., Patrocinio de Oliveira C.E., Candia-Luján R., de Paz J.A. (2015). Métodos de Evaluación de la Composición Corporal: Una Revisión Actualizada de Descripción, Aplicación, Ventajas y Desventajas. Arch. Med. Deporte.

[48] TANITA Corporation Instruction Manual BC-418MA. https://support.tanita.eu/support/solutions/articles/60000955901-instruction-manual-bc-418ma.

[49] Shim J.-S., Oh K., Kim H.C. (2014). Dietary Assessment Methods in Epidemiologic Studies. Epidemiol. Health.

[50] Ingeniería A. DIAL software (Alce Ingeniería, Madrid, Spain) (Ortega RM, López-Sobaler AM, Andrés P, Requejo AM, Aparicio A, Molinero LM. 2013. DIAL software for assessing diets and food calculations (for Windows, version 3.0.0.5). Department of Nutrition (UCM) & Alceingeniería, S.A. Madrid, Spain.

[51] Chisaguano A.M., Lozano B., Moltó-Puigmartí C., Castellote A.I., Rafecas M., López-Sabater M.C. (2013). Elaidic Acid, Vaccenic Acid and Rumenic Acid (c9,t11-CLA) Determination in Human Plasma Phospholipids and Human Milk by Fast Gas Chromatography. Anal. Methods.

[52] Therneau T., Atkinson B. Rpart: Recursive Partitioning and Regression Trees. R Package, version 4.1.27; R package; 2026. https://CRAN.R-project.org/package=rpart.

[53] Strain J., Spaans F., Serhan M., Davidge S.T., Connor K.L. (2022). Programming of Weight and Obesity across the Lifecourse by the Maternal Metabolic Exposome: A Systematic Review. Mol. Asp. Med..

[54] Segovia S.A., Vickers M.H., Gray C., Reynolds C.M. (2014). Maternal Obesity, Inflammation, and Developmental Programming. BioMed Res. Int..

[55] Micallef M., Munro I., Phang M., Garg M. (2009). Plasma n-3 Polyunsaturated Fatty Acids Are Negatively Associated with Obesity. Br. J. Nutr..

[56] Tica S., Luo C., Ren D., Zong X., Thompson M.D., Stoll J., DeBosch B.J., I Tarr P., Cao Y. (2026). Parental Obesity and Risk of Metabolic Dysfunction-Associated Steatotic Liver Disease in Adult Offspring: UK Birth Cohort Study. Gut.

[57] Baack M.L., Puumala S.E., Messier S.E., Pritchett D.K., Harris W.S. (2015). What Is the Relationship between Gestational Age and Docosahexaenoic Acid (DHA) and Arachidonic Acid (ARA) Levels?. Prostaglandins Leukot. Essent. Fat. Acids.

[58] Alvarez D., Muñoz Y., Ortiz M., Maliqueo M., Chouinard-Watkins R., Valenzuela R. (2021). Impact of Maternal Obesity on the Metabolism and Bioavailability of Polyunsaturated Fatty Acids during Pregnancy and Breastfeeding. Nutrients.

[59] Simopoulos A.P. (2016). An Increase in the Omega-6/Omega-3 Fatty Acid Ratio Increases the Risk for Obesity. Nutrients.

[60] Albrecht M., Worthmann A., Heeren J., Diemert A., Arck P.C. (2025). Maternal Lipids in Overweight and Obesity: Implications for Pregnancy Outcomes and Offspring’s Body Composition. Semin. Immunopathol..

[61] Zhang C.X.W., Candia A.A., Sferruzzi-Perri A.N. (2024). Placental Inflammation, Oxidative Stress, and Fetal Outcomes in Maternal Obesity. Trends Endocrinol. Metab..

[62] Young I.E., Parker H.M., Cook R.L., O’Dwyer N.J., Garg M.L., Steinbeck K.S., Cheng H.L., Donges C., Franklin J.L., O’Connor H.T. (2020). Association between Obesity and Omega-3 Status in Healthy Young Women. Nutrients.

[63] Mingay E., Veysey M., Lucock M., Niblett S., King K., Patterson A., Garg M. (2016). Sex-Dependent Association between Omega-3 Index and Body Weight Status in Older Australians. J. Nutr. Intermed. Metab..

[64] Burrows T., Collins C.E., Garg M.L. (2011). Omega-3 Index, Obesity and Insulin Resistance in Children. Int. J. Pediatr. Obes..

[65] Scaglioni S., Verduci E., Salvioni M., Bruzzese M.G., Radaelli G., Zetterström R., Riva E., Agostoni C. (2006). Plasma Long-Chain Fatty Acids and the Degree of Obesity in Italian Children. Acta Paediatr..

[66] Karlsson M., Mårild S., Brandberg J., Lönn L., Friberg P., Strandvik B. (2006). Serum Phospholipid Fatty Acids, Adipose Tissue, and Metabolic Markers in Obese Adolescents. Obesity.

[67] Couet C., Delarue J., Ritz P., Antoine J.M., Lamisse F. (1997). Effect of Dietary Fish Oil on Body Fat Mass and Basal Fat Oxidation in Healthy Adults. Int. J. Obes..

[68] Singla P., Bardoloi A., Parkash A.A. (2010). Metabolic Effects of Obesity: A Review. World J. Diabetes.

[69] Cazzola R., Rondanelli M., Trotti R., Cestaro B. (2011). Effects of Weight Loss on Erythrocyte Membrane Composition and Fluidity in Overweight and Moderately Obese Women. J. Nutr. Biochem..

[70] Rodríguez-Rodríguez P., Ramiro-Cortijo D., Reyes-Hernández C.G., López de Pablo A.L., González M.C., Arribas S.M. (2018). Implication of Oxidative Stress in Fetal Programming of Cardiovascular Disease. Front. Physiol..

[71] Alsharari Z.D., Risérus U., Leander K., Sjögren P., Carlsson A.C., Vikström M., Laguzzi F., Gigante B., Cederholm T., De Faire U. (2017). Serum Fatty Acids, Desaturase Activities and Abdominal Obesity—A Population-Based Study of 60-Year-Old Men and Women. PLoS ONE.

[72] Haugaard S.B., Madsbad S., Høy C.E., Vaag A. (2006). Dietary Intervention Increases n-3 Long-Chain Polyunsaturated Fatty Acids in Skeletal Muscle Membrane Phospholipids of Obese Subjects. Implications for Insulin Sensitivity. Clin. Endocrinol..

[73] Lillycrop K.A., Burdge G.C. (2012). Epigenetic Mechanisms Linking Early Nutrition to Long Term Health. Best Pract. Res. Clin. Endocrinol. Metab..

[74] Vinding R.K., Sevelsted A., Horner D., Vahman N., Lauritzen L., Hagen C.P., Chawes B., Stokholm J., Bønnelykke K. (2024). Fish Oil Supplementation during Pregnancy, Anthropometrics, and Metabolic Health at Age Ten: A Randomized Clinical Trial. Am. J. Clin. Nutr..

[75] Gil-Campos M., Ramírez-Tortosa M.C., Larqué E., Linde J., Aguilera C.M., Cañete R., Gil A. (2008). Metabolic Syndrome Affects Fatty Acid Composition of Plasma Lipids in Obese Prepubertal Children. Lipids.

[76] Takahashi Y., Ide T., Fujita H. (2000). Dietary Gamma-Linolenic Acid in the Form of Borage Oil Causes Less Body Fat Accumulation Accompanying an Increase in Uncoupling Protein 1 mRNA Level in Brown Adipose Tissue. Comp. Biochem. Physiol. B Biochem. Mol. Biol..

[77] Mustonen A.-M., Nieminen P. (2023). Dihomo-γ-Linolenic Acid (20:3n-6)—Metabolism, Derivatives, and Potential Significance in Chronic Inflammation. Int. J. Mol. Sci..

[78] George A.D., Wang T., Duong T., Schooneveldt Y., Paul S., Olshansky G., Mansell T., Saffery R., Vuillermin P., Ponsonby A.-L. (2026). Lipid Pathways Connecting Maternal BMI with Infant Obesity Risk. Sci. Rep..

[79] Ladino L., Demmelmair H., Segura M.T., Escudero-Marin M., Grote V., Koletzko B., Campoy C. (2025). Association Between Maternal Dietary Fatty Acid Intake and Fatty Acid Composition of Placental Phospholipids. Nutrients.

[80] Lee J.-E., Ge K. (2014). Transcriptional and Epigenetic Regulation of PPARγ Expression during Adipogenesis. Cell Biosci..

[81] Banaszak M., Górna I., Drzymała-Czyż S. (2025). Fatty Acid Profile and Desaturase Activity in Obesity: Roles, Mechanisms, and Clinical Relevance. Metabolites.

[82] Lager S., Ramirez V.I., Gaccioli F., Jang B., Jansson T., Powell T.L. (2016). Protein Expression of Fatty Acid Transporter 2 Is Polarized to the Trophoblast Basal Plasma Membrane and Increased in Placentas from Overweight/Obese Women. Placenta.

[83] Fujimori K. (2012). Prostaglandins as PPARγ Modulators in Adipogenesis. PPAR Res..

[84] Vidakovic A.J., Gishti O., Voortman T., Felix J.F., Williams M.A., Hofman A., Demmelmair H., Koletzko B., Tiemeier H., Jaddoe V.W.V. (2016). Maternal Plasma PUFA Concentrations during Pregnancy and Childhood Adiposity: The Generation R Study. Am. J. Clin. Nutr..

[85] Zembura M., Matusik P. (2022). Sarcopenic Obesity in Children and Adolescents: A Systematic Review. Front. Endocrinol..

[86] Donini L.M., Busetto L., Bischoff S.C., Cederholm T., Ballesteros-Pomar M.D., Batsis J.A., Bauer J.M., Boirie Y., Cruz-Jentoft A.J., Dicker D. (2022). Definition and Diagnostic Criteria for Sarcopenic Obesity: ESPEN and EASO Consensus Statement. Clin. Nutr..

[87] Sessler A.M., Ntambi J.M. (1998). Polyunsaturated Fatty Acid Regulation of Gene Expression. J. Nutr..

[88] Marchix J., Alain C., David-Le Gall S., Acuña-Amador L.A., Druart C., Delzenne N.M., Barloy-Hubler F., Legrand P., Boudry G. (2020). Maternal Linoleic Acid Overconsumption Alters Offspring Gut and Adipose Tissue Homeostasis in Young but Not Older Adult Rats. Nutrients.

[89] Heerwagen M.J.R., Stewart M.S., de la Houssaye B.A., Janssen R.C., Friedman J.E. (2013). Transgenic Increase in N-3/N-6 Fatty Acid Ratio Reduces Maternal Obesity-Associated Inflammation and Limits Adverse Developmental Programming in Mice. PLoS ONE.

[90] Herrera E., Ortega-Senovilla H. (2023). Dietary Implications of Polyunsaturated Fatty Acids during Pregnancy and in Neonates. Life.

[91] Desoye G., Schweditsch M.O., Pfeiffer K.P., Zechner R., Kostner G.M. (1987). Correlation of Hormones with Lipid and Lipoprotein Levels during Normal Pregnancy and Postpartum. J. Clin. Endocrinol. Metab..

[92] Shokry E., Marchioro L., Uhl O., Bermúdez M.G., García-Santos J.A., Segura M.T., Campoy C., Koletzko B. (2019). Transgenerational Cycle of Obesity and Diabetes: Investigating Possible Metabolic Precursors in Cord Blood from the PREOBE Study. Acta Diabetol..

[93] Palacios S., Cancelo M.J., Castaño M.R., García A., de la Gándara J.J., Pintó X., Sánchez Borrego R., Bannenberg G., Gil E. (2014). Recomendaciones de Ingesta de Omega 3 en los Diferentes Periodos de la Vida de la Mujer. Prog. Obstet. Ginecol..

[94] Livingstone M.B.E., Robson P.J., Wallace J.M.W. (2004). Issues in Dietary Intake Assessment of Children and Adolescents. Br. J. Nutr..

[95] Murakami K., Livingstone M.B.E., Okubo H., Sasaki S. (2016). Younger and Older Ages and Obesity Are Associated with Energy Intake Underreporting but Not Overreporting in Japanese Boys and Girls Aged 1–19 Years: The National Health and Nutrition Survey. Nutr. Res..

[96] Poppitt S.D., Swann D., Black A.E., Prentice A.M. (1998). Assessment of Selective Under-Reporting of Food Intake by Both Obese and Non-Obese Women in a Metabolic Facility. Int. J. Obes..

[97] Parisi F., Mandò C., Novielli C., Anelli G.M., Cazzola R., Lisso F., Sarno L., Marelli E., Lubrano C., Antonazzo P.G.M. (2025). Maternal Mid-Pregnancy Long-Chain Polyunsaturated Fatty Acid Profile Is Associated with Pregestational Body Mass Index and Neonatal Anthropometric Measures at Birth among Non-Obese Pregnancies: Results from Two Italian Multicenter Cohorts. Nutr. Metab..

[98] White P.A., Awad Y.A., Gauvin L., Spencer N.J., McGrath J.J., Clifford S.A., Nikiema B., Yang-Huang J., Goldhaber-Fiebert J.D., Markham W. (2022). Household Income and Maternal Education in Early Childhood and Risk of Overweight and Obesity in Late Childhood: Findings from Seven Birth Cohort Studies in Six High-Income Countries. Int. J. Obes..

[99] Shrestha N., Sleep S.L., Cuffe J.S.M., Holland O.J., Perkins A.V., Yau S.Y., McAinch A.J., Hryciw D.H. (2020). Role of Omega-6 and Omega-3 Fatty Acids in Fetal Programming. Clin. Exp. Pharmacol. Physiol..

[100] Homs C., Berruezo P., Arcarons A., Wärnberg J., Osés M., González-Gross M., Gusi N., Aznar S., Marín-Cascales E., González-Valeiro M.Á. (2023). Independent and Joined Association between Socioeconomic Indicators and Pediatric Obesity in Spain: The PASOS Study. Nutrients.

[101] Miqueleiz E., Lostao L., Ortega P., Santos J.M., Astasio P., Regidor E. (2014). Trends in the Prevalence of Childhood Overweight and Obesity According to Socioeconomic Status: Spain, 1987–2007. Eur. J. Clin. Nutr..

[102] Goldstein R.F., Abell S.K., Ranasinha S., Misso M., Boyle J.A., Black M.H., Li N., Hu G., Corrado F., Rode L. (2017). Association of Gestational Weight Gain with Maternal and Infant Outcomes: A Systematic Review and Meta-Analysis. JAMA.

[103] Solmi F., Morris S. (2015). Association between Childhood Obesity and Use of Regular Medications in the UK: Longitudinal Cohort Study of Children Aged 5–11 Years. BMJ Open.

[104] Rudolph M.C., Jackman M.R., Presby D.M., Houck J.A., Webb P.G., Johnson G.C., Soderborg T.K., de la Houssaye B.A., Yang I.V., Friedman J.E. (2018). Low Neonatal Plasma n-6/n-3 PUFA Ratios Regulate Offspring Adipogenic Potential and Condition Adult Obesity Resistance. Diabetes.

[105] Maslova E., Rifas-Shiman S.L., Olsen S.F., Gillman M.W., Oken E. (2018). Prenatal n-3 Long-Chain Fatty Acid Status and Offspring Metabolic Health in Early and Mid-Childhood: Results from Project Viva. Nutr. Diabetes.

[106] Donahue S.M., Rifas-Shiman S.L., Gold D.R., Jouni Z.E., Gillman M.W., Oken E. (2011). Prenatal Fatty Acid Status and Child Adiposity at Age 3 y: Results from a US Pregnancy Cohort. Am. J. Clin. Nutr..

[107] Xue M., Liu S., Zhang X., Zhang Z., Niu W. (2026). Development and External Validation of an Interpretable Machine Learning-Based Model for Obesity Risk Prediction in 2–18-Year-Old Children and Adolescents in Beijing and Tangshan. J. Glob. Health.

[108] Heindel J.J., Alvarez J.A., Atlas E., Cave M.C., Chatzi V.L., Collier D., Corkey B., Fischer D., Goran M.I., Howard S. (2023). Obesogens and Obesity: State-of-the-Science and Future Directions Summary from a Healthy Environment and Endocrine Disruptors Strategies Workshop. Am. J. Clin. Nutr..

[109] Ulug E., Nergiz-Unal R. (2021). Dietary Fatty Acids and CD36-Mediated Cholesterol Homeostasis: Potential Mechanisms. Nutr. Res. Rev..

[110] Kashyap M.L., Sivasamboo R., Sothy S.P., Cheah J.S., Gartside P.S. (1976). Carbohydrate and Lipid Metabolism during Human Labor: Free Fatty Acids, Glucose, Insulin, and Lactic Acid Metabolism during Normal and Oxytocin-Induced Labor for Postmaturity. Metabolism.

[111] Muir R., Liu G., Khan R., Shmygol A., Quenby S., Gibson R.A., Muhlhausler B., Elmes M. (2018). Maternal Obesity-Induced Decreases in Plasma, Hepatic and Uterine Polyunsaturated Fatty Acids during Labour Is Reversed through Improved Nutrition at Conception. Sci. Rep..

[112] HAPO Study Cooperative Research Group (2009). Hyperglycemia and Adverse Pregnancy Outcome (HAPO) Study: Associations with Neonatal Anthropometrics. Diabetes.

