# Supplementary material for: Maternal Overweight/Obesity and Polyunsaturated Fatty Acid Status During Pregnancy Are Associated with Offspring Body Composition at 8.5 Years: Findings from the PREOBE Study
Source: Nutrients. 2026 Oct 21;18(20):3453. doi: 10.3390/nu18203453 (PMC13708055; doi:10.3390/nu18203453)
Supplement: Supplementary file 1 [file nutrients-18-03453-s001.zip › nutrients-4452621-supplementary.pdf]

Manuscript nutrients-4452621: *"Maternal Overweight/Obesity and Polyunsaturated Fatty Acid Status During Pregnancy are Associated with Offspring Body Composition at 8.5 Years: Findings from the PREOBE Study"*

Supplementary Material (2nd revision)

Supplementary Table S1: Daily intake reported by mothers at 34 weeks of pregnancy (n=147), depending on maternal pre-gestational BMI.

Daily reported intake	NW (n=73)	OW (n=40)	OB (n=34)	P-value
Kcal/day	2078.33 ± 39.49[a]	2159.74 ± 99.04[a]	1800.85 ± 82.4[b]	**0.043#**
Carbohydrates (g)	224.00 ± 5.76[a]	231.89 ± 8.08[a]	180.47 ± 9.55[b]	**0.004**
Sugar (g)	82.43 ± 1.58	86.06 ± 4.00	71.51 ± 3.24	**0.042#**
Proteins (g)	85.30 ± 2.07	90.11 ± 3.49	76.43 ± 3.32	0.087
Lipids (g)	87.11 ± 2.29	91.34 ± 7.05	80.66 ± 3.81	0.511
Cholesterol (mg)	296.72 ± 9.36	326.63 ± 26	293.90 ± 10.99	0.499
SFA (g)	30.85 ± 0.98	32.79 ± 3.18	28.43 ± 1.39	0.558
MUFA (g)	36.78 ± 1.14	37.15 ± 2.67	32.88 ± 1.77	0.474
PUFA (g)	12.13 ± 0.39	13.61 ± 0.98	12.34 ± 0.72	0.412
LA (g)	10.00 ± 0.34	11.41 ± 0.86	10.27 ± 0.63	0.362
ARA (g)	0.14 ± 0.01	0.18 ± 0.02	0.15 ± 0.01	0.128
PUFA n6 (g)	10.43 ± 0.35	11.94 ± 0.88	10.77 ± 0.61	0.164
ALA (g)	1.23 ± 0.05	1.32 ± 0.08	1.08 ± 0.06	0.213
EPA (g)	0.14 ± 0.01	0.09 ± 0.02	0.15 ± 0.02	0.164
DPA n3 (g)	0.04 ± 0 .00	0.04 ± 0.00	0.04 ± 0.01	0.903
DHA (g)	0.29 ± 0.02	0.22 ± 0.03	0.30 ± 0.04	0.288
PUFA n3 (g)	1.70 ± 0.05	1.67 ± 0.09	1.57± 0.08	0.438
**Indexes**				
PUFA n6/n3	6.14 ± 0.29	7.15 ± 0.66	6.86 ± 0.53	0.233
LC-PUFA n6/n3	0.31 ± 0.03a	0.52 ± 0.08b	0.32 ± 0.04a	**0.002**
LA/ALA	8.13 ± 0.25	8.65 ± 0.35	9.51 ± 0.49	0.161
ARA/DHA	0.48 ± 0.05a	0.82 ± 0.14b	0.50 ± 0.07a	**0.010**
ARA/DHA+EPA	0.33 ± 0.03a	0.58 ± 0.09b	0.33 ± 0.04a	**0.002**

Normal-weight (NW), overweight (OW) or obese (OB) pregnant women. ALA: α-linolenic acid; ARA: arachidonic acid; DHA: docosahexaenoic acid; DPA: docosapentaenoic acid; EPA: eicosapentaenoic acid; LA: linoleic acid; LC-PUFA: long-chain polyunsaturated fatty acids; MUFA: monounsaturated fatty acids; PUFA: polyunsaturated fatty acids; SFA: saturated fatty acids. All data are expressed as Mean ± Standard Error and variables were normally distributed. Statistical analysis performed: ANOVA: Analysis of variance for normally distributed variables. Values who do not share the same suffix (abc) are significantly different in a Bonferroni post hoc test. #Loss of significance after post hoc test. P: Level of significance for overall differences between PREOBE-groups. Bold: p-value ≤ 0.05.

Supplementary Table S2. Children's dietary intake at 8.5 years old (n =147), classified depending on their maternal pre-gestational BMI group.

Daily reported intake	NW (n=73)	OW (n=40)	OB (n=34)	P-value
Kcal/day	2043.29 ± 43.06	2055.93±58.73	1856.88 ± 47.12	**0.038**[#]
Carbohydrates (g)	207.06 ± 5.07[a]	210.52 ± 5.93[a]	179.71 ± 6.36[b]	**0.004**
Sugar (g)	99.67 ± 3.37[a]	99.25 ± 3.91[a]	80.03 ± 4.47[b]	**0.004**
Proteins (g)	79.74 ± 1.85	78.18 ± 2.43	73.2 ± 2.07	0.149
Lipids (g)	96.12 ± 2.38	96.66 ± 3.6	90.91 ± 2.65	0.441
Cholesterol (mg)	340.4 ± 9.95	325.68 ± 15.01	300.97 ± 10.95	0.112
SFA (g)	33.58 ± 0.9	33.66 ± 1.42	29.59 ± 0.93	**0.048**[#]
MUFA (g)	43.53 ± 1.21	43.2 ± 1.78	41.53 ± 1.38	0.672
PUFA (g)	10.98 ± 0.39	11.54 ± 0.55	11.57 ± 0.59	0.636
LA (g)	8.74 ± 0.34	9.39 ± 0.5	9.56 ± 0.53	0.320
ARA (g)	0.14 ± 0.01	0.16 ± 0.01	0.16 ± 0.01	0.320
PUFA n6 (g)	9.62±0.35	10.27±0.52	10.14±0.54	0.319
ALA (g)	1.04 ± 0.05	0.97 ± 0.04	1.01 ± 0.06	0.680
EPA (g)	0.07 ± 0.01	0.06 ± 0.02	0.1 ± 0.02	0.347
DPA n3 (g)	0.03 ± 0.00	0.04 ± 0.01	0.04 ± 0.01	0.243
DHA (g)	0.22 ± 0.02	0.2 ± 0.03	0.28 ± 0.04	0.164
PUFA n3 (g)	1.36±0.05	1.27±0.05	1.43±0.08	0.279
**Indexes**				
PUFA n6/n3	7.07±0.37	8.09±0.52	7.09±0.53	0.271
LC-PUFA n6/n3	0.45±0.04	0.55±0.07	0.41±0.05	0.150
LA/ALA	8.40±0.52	9.68±0.65	9.47±0.77	0.257
ARA/DHA	0.64±0.07	0.80±0.13	0.57±0.09	0.300
ARA/DHA+EPA	0.48±0.05	0.62±0.09	0.42±0.06	0.151

8.5-years-old children born to normal weight (NW), overweight (OW) or obese (OB). ALA: α-linolenic acid; ARA: arachidonic acid; DHA: docosahexaenoic acid; DPA: docosapentaenoic acid; EPA: eicosapentaenoic acid; LA: linoleic acid; LC-PUFA: long-chain polyunsaturated fatty acids; MUFA: monounsaturated fatty acids; PUFA: polyunsaturated fatty acids; SFA: saturated fatty acids. All data are expressed as Mean ± Standard Error and variables were normally distributed. Statistical analysis performed: ANOVA: Analysis of variance for normally distributed variables. Values who do not share the same suffix (abc) are significantly different in a Bonferroni post hoc test. #Loss of significance after post hoc test. P: Level of significance for overall differences between PREOBE-groups. Bold: p-value ≤ 0.05.

Supplementary Table S3. Selected correlations between maternal n-3 polyunsaturated fatty acids in plasma phospholipid and offspring anthropometry and body composition at 8.5 years, corresponding to Table 6.

Gestation Week	Fatty Acid (%)	Offspring anthropometry	NW r	NW p	NW q	OW r	OW p	OW q	OB r	OB p	OB q
34	ALA (C18:3n-3)	BAZ	-0.17	0.215	0.409	0.41	**0.030**	**0.036**	-0.12	0.604	0.754
34	ALA (C18:3n-3)	MUACZ	0.18	0.189	0.399	0.67	**<0.001**	**<0.001**	-0.19	0.410	0.708
34	ALA (C18:3n-3)	TSF	0.04	0.772	0.874	0.61	**<0.001**	**<0.001**	-0.08	0.730	0.771
34	ALA (C18:3n-3)	TSFZ	-0.16	0.243	0.420	0.65	**<0.001**	**<0.001**	-0.09	0.698	0.771
34	ALA (C18:3n-3)	"FM (%)[1]"	-0.15	0.274	0.434	0.60	**<0.001**	**<0.001**	-0.11	0.635	0.754
34	ALA (C18:3n-3)	FMI	-0.11	0.424	0.575	0.47	**0.012**	**0.016**	-0.13	0.574	0.754
34	ALA (C18:3n-3)	FMI/FFMI	-0.20	0.143	0.340	0.51	**0.006**	**0.011**	-0.19	0.410	0.708
34	ALA (C18:3n-3)	"TFFM (kg)[2]"	0.22	0.107	0.290	-0.52	**0.005**	**0.011**	0.05	0.830	0.830
34	ALA (C18:3n-3)	"Trunk FM (%)[2]"	-0.03	0.828	0.874	0.62	**<0.001**	**<0.001**	-0.11	0.635	0.754
40	ALA (C18:3n-3)	HAZ	0.49	**<0.001**	**0.001**	-0.42	**0.026**	**0.033**	-0.39	0.081	0.385
40	ALA (C18:3n-3)	"FFM (kg)[1]"	0.41	**0.002**	**0.013**	-0.49	**0.008**	**0.014**	-0.19	0.410	0.708
40	ALA (C18:3n-3)	FFMI	0.02	0.885	0.885	0.10	0.613	0.647	0.51	0.019	0.228
40	ALA (C18:3n-3)	"FM (%)[2]"	0.14	0.308	0.450	-0.57	**0.002**	**0.006**	0.23	0.316	0.708
40	ALA (C18:3n-3)	"TFFM (kg)[2]"	0.09	0.513	0.650	0.48	**0.010**	**0.016**	-0.19	0.410	0.708
34	DPA (C22:5n-3)	FFMI	0.03	0.828	0.874	0.12	0.543	0.607	0.49	0.024	0.228
34	DPA (C22:5n-3)	"Trunk FM (%)[2]"	0.22	0.107	0.290	0.54	**0.003**	**0.008**	-0.31	0.171	0.650
40	DPA (C22:5n-3)	WAZ	0.37	**0.005**	**0.019**	0.02	0.920	0.920	-0.20	0.385	0.708
40	DPA (C22:5n-3)	HAZ	0.53	**<0.001**	**<0.001**	-0.47	**0.012**	**0.016**	-0.40	0.072	0.385
40	DPA (C22:5n-3)	"TFFM (kg)[2]"	0.39	**0.003**	**0.014**	-0.52	**0.005**	**0.011**	-0.16	0.488	0.754

ALA, α-linolenic acid; DPA, docosapentaenoic acid; BAZ, BMI-for-age z-score; FFM, fat-free mass; FFMI, fat-free mass index; FM, fat mass; FMI, fat mass index; HAZ, height-for-age z-score; MUACZ, mid-upper-arm circumference-for-age z-score; NW, normal weight; OW, overweight; OB, obesity; TFFM, total fat-free mass; TSF, triceps skinfold; TSFZ, triceps skinfold-for-age z-score; WAZ, weight-for-age z-score. [1]Estimated using the Slaughter equations; [2]estimated using bioelectrical impedance analysis (TANITA®). Fatty acids are expressed as relative % of total plasma phospholipids measured; r denotes the reported Spearman rank correlation coefficient; p denotes the nominal two-sided p-value; q denotes the reported FDR-adjusted value. The displayed adjustment set contains 19 comparisons within each BMI group. Bold formatting identifies comparisons reported with q < 0.05. These unadjusted subgroup correlations do not establish confounder-independent associations or differences between subgroup effects.

Supplementary Table S4. Selected correlations between maternal or umbilical cord n-6 polyunsaturated fatty acids or fatty-acid ratios in plasma phospholipid and offspring anthropometry and body composition at 8.5 years, corresponding to Table 7.

Gestation week	Fatty Acid (%)	Offspring anthropometry	NW r	NW p	NW q	OW r	OW p	OW q	OB r	OB p	OB q
24	LA (C18:2n-6)	TSF	-0.09	0.513	0.581	0.54	**0.003**	**0.036**	0.06	0.796	0.990
24	LA (C18:2n-6)	TSFZ	-0.23	0.088	0.122	0.49	**0.008**	**0.036**	0.02	0.931	1.000
24	LA (C18:2n-6)	"FM (%)[1]"	-0.27	0.042	0.065	0.50	**0.007**	**0.036**	-0.01	0.966	1.000
24	LA (C18:2n-6)	FMI	0.05	0.717	0.734	0.50	**0.007**	**0.036**	-0.01	0.966	1.000
24	LA (C18:2n-6)	FFMI	-0.29	0.031	0.056	0.53	**0.004**	**0.036**	-0.04	0.863	1.000
24	AdA (C22:4n-6)	"TFFM (kg)[2]"	-0.08	0.561	0.619	0.41	0.030	0.059	-0.06	0.796	0.990
24	AdA (C22:4n-6)	FMI/FFMI	0.09	0.513	0.581	-0.40	0.035	0.063	0.07	0.763	0.990
34	LA (C18:2n-6)	MUACZ	0.31	**0.021**	**0.039**	0.05	0.801	0.883	-0.06	0.796	0.990
34	GLA (C18:3n-6)	MUACZ	-0.28	0.038	0.061	0.08	0.686	0.894	0.06	0.796	0.990
34	EDA (C20:2n-6)	"MUAC (cm)"	0.59	**<0.001**	**<0.001**	-0.17	0.387	0.594	-0.16	0.488	0.990
34	EDA (C20:2n-6)	MUACZ	0.60	**<0.001**	**<0.001**	-0.23	0.239	0.381	-0.19	0.410	0.990
34	ARA (C20:4n-6)	WAZ	0.38	**0.004**	**0.009**	0.03	0.880	0.923	0.14	0.545	0.990
34	ARA (C20:4n-6)	"BFFM (%)[2]"	-0.17	0.215	0.272	-0.47	**0.012**	**0.037**	0.11	0.635	0.990
34	ARA (C20:4n-6)	"BFFM (kg)[2]"	-0.19	0.163	0.219	-0.48	**0.010**	**0.036**	0.12	0.604	0.990
34	AdA (C22:4n-6)	"FFM (kg)[2]"	0.15	0.274	0.337	-0.51	**0.006**	**0.036**	0.13	0.574	0.990
40	GLA (C18:3n-6)	"Height (cm)"	-0.28	0.038	0.061	0.06	0.762	0.910	0.09	0.698	0.990
40	GLA (C18:3n-6)	HAZ	-0.31	**0.021**	**0.039**	0.02	0.920	0.920	0.09	0.698	0.990
40	PUFAs n-6/n-3 ratio	FMI/FFMI	0.09	0.513	0.581	-0.09	0.649	0.930	0.44	0.046	0.989
UC	GLA (C18:3n-6)	"Weight (kg)"	-0.45	**<0.001**	**<0.001**	-0.07	0.723	0.914	0.10	0.666	0.990
UC	GLA (C18:3n-6)	WAZ	-0.47	**<0.001**	**<0.001**	-0.09	0.649	0.930	0.13	0.574	0.990
UC	GLA (C18:3n-6)	"WC (cm)"	-0.48	**<0.001**	**<0.001**	-0.46	**0.014**	**0.040**	-0.04	0.863	1.000
UC	GLA (C18:3n-6)	WCZ	-0.50	**<0.001**	**<0.001**	-0.45	**0.016**	**0.043**	-0.01	0.966	1.000
UC	GLA (C18:3n-6)	WHR	-0.34	**0.011**	**0.024**	-0.36	0.060	0.099	-0.19	0.410	0.990
UC	GLA (C18:3n-6)	BAZ	-0.46	**<0.001**	**<0.001**	-0.40	0.035	0.063	-0.01	0.966	1.000
UC	GLA (C18:3n-6)	TSFZ	-0.55	**<0.001**	**<0.001**	-0.42	0.026	0.053	-0.11	0.635	0.990
UC	GLA (C18:3n-6)	"FM (%)[1]"	-0.48	**<0.001**	**<0.001**	-0.02	0.920	0.920	-0.02	0.931	1.000
UC	GLA (C18:3n-6)	"FM (kg)[1]"	-0.51	**<0.001**	**<0.001**	-0.44	**0.019**	**0.045**	-0.03	0.897	1.000
UC	GLA (C18:3n-6)	"FFM (%)[1]"	-0.38	**0.004**	**0.009**	-0.05	0.801	0.883	0.27	0.237	1.000

UC	GLA (C18:3n-6)	"FFM (kg)[1]"	-0.41	**0.001**	**0.003**	-0.04	0.840	0.903	0.05	0.830	1.000
UC	GLA (C18:3n-6)	FFMI	-0.31	**0.018**	**0.037**	-0.43	**0.022**	**0.047**	-0.52	0.016	0.688
UC	GLA (C18:3n-6)	FMI/FFMI	-0.44	**<0.001**	**<0.001**	-0.49	**0.008**	**0.036**	-0.05	0.830	1.000
UC	GLA (C18:3n-6)	"BFM (%)[2]"	-0.49	**<0.001**	**<0.001**	-0.06	0.762	0.910	-0.16	0.488	0.990
UC	GLA (C18:3n-6)	"BFM (kg)[2]"	-0.44	**<0.001**	**<0.001**	-0.05	0.801	0.883	0.09	0.698	0.990
UC	GLA (C18:3n-6)	"BFFM (%)[2]"	-0.39	**0.003**	**0.008**	-0.08	0.686	0.894	0.08	0.730	0.990
UC	GLA (C18:3n-6)	"BFFM (kg)[2]"	-0.45	**<0.001**	**<0.001**	-0.48	**0.010**	**0.036**	-0.003	0.990	1.000
UC	GLA (C18:3n-6)	"BTrunkFM (%)[2]"	-0.52	**<0.001**	**<0.001**	-0.43	**0.022**	**0.047**	-0.08	0.730	0.990
UC	DGLA (C20:3n-6)	"FM (kg)[2]"	-0.01	0.942	0.942	-0.49	**0.008**	**0.036**	0.06	0.796	0.990
UC	ARA (C20:4n-6)	BAZ	-0.17	0.214	0.272	0.55	**0.002**	**0.036**	0.07	0.763	0.990
UC	ARA (C20:4n-6)	FFMI	0.07	0.612	0.642	-0.08	0.686	0.894	-0.28	0.219	1.000
UC	ARA (C20:4n-6)	FMI/FFMI	-0.23	0.088	0.122	0.47	**0.012**	**0.037**	0.09	0.698	0.990
UC	AdA (C22:4n-6)	BAZ	0.07	0.612	0.642	-0.48	**0.010**	**0.036**	0.06	0.796	0.990
UC	AdA (C22:4n-6)	"BFFM (kg)[2]"	-0.25	0.062	0.092	0.44	**0.019**	**0.045**	0.02	0.931	1.000
UC	AdA (C22:4n-6)	FMI/FFMI	0.28	0.038	0.061	-0.38	0.046	0.079	-0.10	0.666	0.990

AdA, adrenic acid; ARA, arachidonic acid; DGLA, dihomo-γ-linolenic acid; EDA, eicosadienoic acid; GLA, γ-linolenic acid; LA, linoleic acid; BAZ, BMI-for-age z-score; FFM, fat-free mass; FFMI, fat-free mass index; FM, fat mass; FMI, fat mass index; HAZ, height-for-age z-score; MUAC, mid-upper-arm circumference; MUACZ, mid-upper-arm circumference-for-age z-score; NW, normal weight; OW, overweight; OB, obesity; PUFA, polyunsaturated fatty acid; TFFM, total fat-free mass; TSF, triceps skinfold; TSFZ, triceps skinfold-for-age z-score; UC: Umbilical cord; WAZ, weight-for-age z-score; WC, waist circumference; WCZ, waist circumference-for-age z-score; WHR, waist-to-hip ratio. [1]Estimated using the Slaughter equations; [2]estimated using bioelectrical impedance analysis (TANITA®). Fatty acids are expressed as relative % of total plasma phospholipids measured; r denotes the reported Spearman rank correlation coefficient; p denotes the nominal two-sided p-value; q denotes the reported FDR-adjusted value. The displayed adjustment set contains 43 comparisons within each BMI group. Bold formatting identifies comparisons reported with $q < 0.05$. These unadjusted subgroup correlations do not establish confounder-independent associations or differences between subgroup effects.
